# Supplementary material for: New Series of Double-Modified Colchicine Derivatives: Synthesis, Cytotoxic Effect and Molecular Docking
Source: Molecules. 2020 Aug 2;25(15):3540. doi: 10.3390/molecules25153540 (PMC7435877; doi:10.3390/molecules25153540)
Supplement: Supplementary file 1 [file molecules-25-03540-s001.pdf]

New Series of Double-Modified Colchicine Derivatives: Synthesis, Cytotoxic Effect and Molecular Docking

Julia Krzywik [1,2], Maral Aminpour [3], Ewa Maj [4], Witold Mozga [2], Joanna Wietrzyk [4], Jack A. Tuszyński [3,5], Adam Huczyński [2,*]

[1] Department of Medical Chemistry, Faculty of Chemistry, Adam Mickiewicz University, Uniwersytetu Poznańskiego 8, 61–614 Poznań, Poland; julia.krzywik@amu.edu.pl (J.K.), adhucz@amu.edu.pl (A.H.)

[2] TriMen Chemicals, Piłsudskiego 141, 92–318 Łódź, Poland; mozga@trimen.pl

[3] Department of Oncology, University of Alberta, Edmonton, T6G 1Z2, Canada; aminpour@ualberta.ca (M.A.); jack.tuszynski@gmail.com (J.A.T.)

[4] Hirszfeld Institute of Immunology and Experimental Therapy, Polish Academy of Sciences, Rudolfa Weigla 12, 53–114 Wrocław, Poland; ewa.maj@hirszfeld.pl (E.M.), joanna.wietrzyk@hirszfeld.pl (J.W.)

[5] DIMEAS, Politecnico di Torino, Corso Duca degli Abruzzi, 24, 10129 Torino, Italy

Supplementary Materials

Table S1. Computational predictions of interactions between tested compounds (**1–17**) and homology modeled tubulin βI.

MATERIALS AND METHODS

General

Spectroscopic measurements

Synthesis of 2 and 3

***In vitro* antiproliferative activity**

Molecular docking studies

LC-MS chromatograms and mass spectra, ^{1}H NMR and ^{13}C NMR spectra of compounds 2-17.

Figure S1. The LC-MS chromatogram and mass spectra of **2**.
Figure S2. The ^{1}H NMR spectrum of **2** in $CDCl_3$.
Figure S3. The ^{13}C NMR spectrum of **2** in $CDCl_3$.
Figure S4. The LC-MS chromatogram and mass spectra of **3**.
Figure S5. The ^{1}H NMR spectrum of **3** in $CDCl_3$.
Figure S6. The ^{13}C NMR spectrum of **3** in $CDCl_3$.
Figure S7. The LC-MS chromatogram and mass spectra of **4**.
Figure S8. The LC-MS chromatogram and mass spectra of **5**.
Figure S9. The ^{1}H NMR spectrum of **5** in $CDCl_3$.

Figure S10. The ^{13}C NMR spectrum of **5** in $CDCl_3$.
Figure S11. The LC-MS chromatogram and mass spectra of **6**.
Figure S12. The ^{1}H NMR spectrum of **6** in $CDCl_3$.
Figure S13. The ^{13}C NMR spectrum of **6** in $CDCl_3$.
Figure S14. The LC-MS chromatogram and mass spectra of **7**.
Figure S15. The ^{1}H NMR spectrum of **7** in $CDCl_3$.
Figure S16. The ^{13}C NMR spectrum of **7** in $CDCl_3$.
Figure S17. The LC-MS chromatogram and mass spectra of **8**.
Figure S18. The ^{1}H NMR spectrum of **8** in $CDCl_3$.
Figure S19. The ^{13}C NMR spectrum of **8** in $CDCl_3$.
Figure S20. The LC-MS chromatogram and mass spectra of **9**.
Figure S21. The ^{1}H NMR spectrum of **9** in $CDCl_3$.
Figure S22. The ^{13}C NMR spectrum of **9** in $CDCl_3$.
Figure S23. The LC-MS chromatogram and mass spectra of **10**.
Figure S24. The ^{1}H NMR spectrum of **10** in $CDCl_3$.
Figure S25. The LC-MS chromatogram and mass spectra of **11**.
Figure S26. The ^{1}H NMR spectrum of **11** in CD_2Cl_2.
Figure S27. The ^{13}C NMR spectrum of **11** in CD_2Cl_2.
Figure S28. The LC-MS chromatogram and mass spectra of **12**.
Figure S29. The ^{1}H NMR spectrum of **12** in $CDCl_3$.
Figure S30. The ^{13}C NMR spectrum of **12** in $CDCl_3$.
Figure S31. The LC-MS chromatogram and mass spectra of **13**.
Figure S32. The ^{1}H NMR spectrum of **13** in $CDCl_3$.
Figure S33. The ^{13}C NMR spectrum of **13** in $CDCl_3$.
Figure S34. The LC-MS chromatogram and mass spectra of **14**.
Figure S35. The ^{1}H NMR spectrum of **14** in $CDCl_3$.
Figure S36. The ^{13}C NMR spectrum of **14** in $CDCl_3$.
Figure S37. The LC-MS chromatogram and mass spectra of **15**.
Figure S38. The ^{1}H NMR spectrum of **15** in $CDCl_3$.
Figure S39. The ^{13}C NMR spectrum of **15** in $CDCl_3$.
Figure S40. The LC-MS chromatogram and mass spectra of **16**.
Figure S41. The ^{1}H NMR spectrum of **16** in CD_3CN.
Figure S42. The ^{13}C NMR spectrum of **16** in CD_3CN.
Figure S43. The LC-MS chromatogram and mass spectra of **17**.
Figure S44. The ^{1}H NMR spectrum of **17** in $CDCl_3$.
Figure S45. The ^{13}C NMR spectrum of **17** in $CDCl_3$.

Table S1. Computational predictions of interactions between tested compounds (**1–17**) and homology modeled tubulin βI

3D representation and 2D layout of colchicine derivatives–tubulin protein complex, binding energy and active residues are tabulated.

Compound	3D representation of the interactions	2D representation of the interactions	Binding Energy [kcal/mol]	Active residues
1	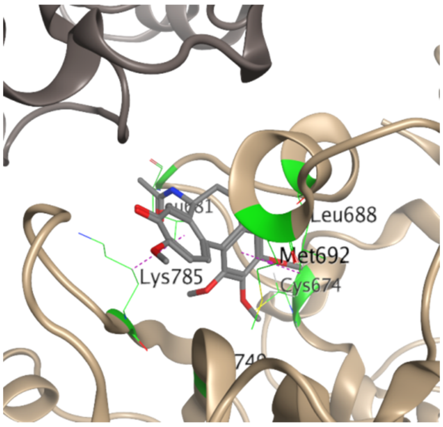	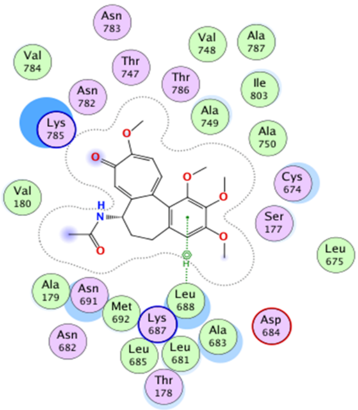	-39.1	**Cys674**<br>Leu681<br>Ala683<br>**Leu688**<br>Met692<br>Ala749<br>**Lys785**
2	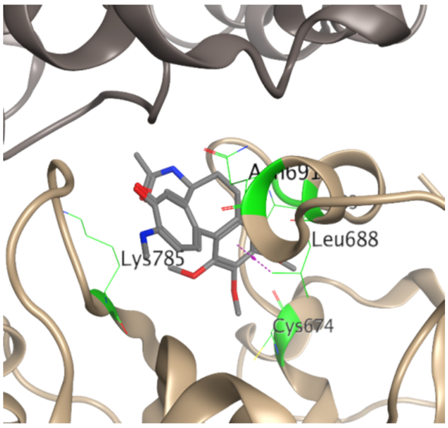	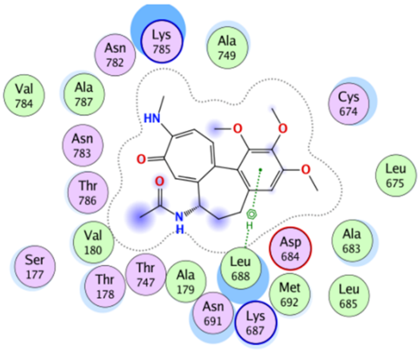	-37.5	Cys674<br>Ala683<br>**Leu688**<br>Asn691<br>**Lys785**
3	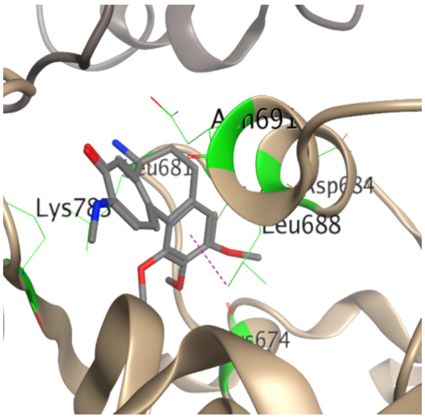	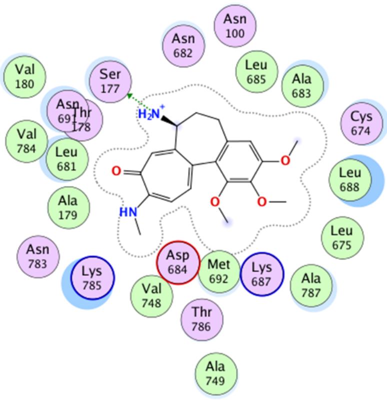	-4.5	**Ser177**<br>Cys674<br>**Leu681**<br>Ala683<br>Asp684<br>**Leu688**<br>Asn691<br>Lys785

4	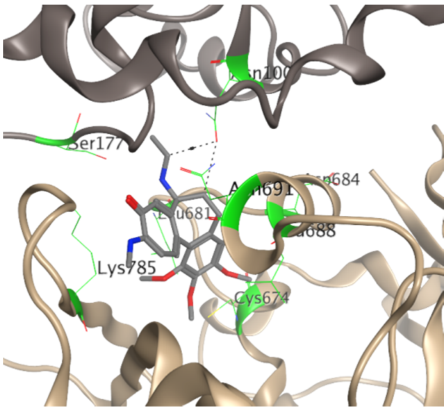	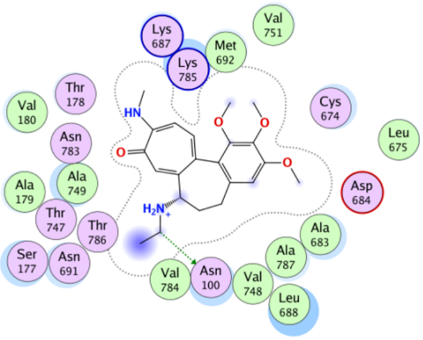	-17.2	Asn100<br>**Ser177**<br>Cys674<br>Leu681<br>Asp684<br>**Leu688**<br>Asn691<br>**Lys785**
5	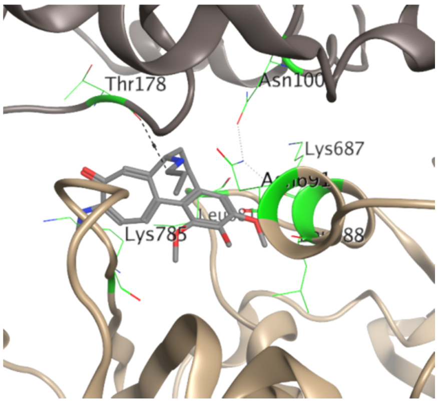	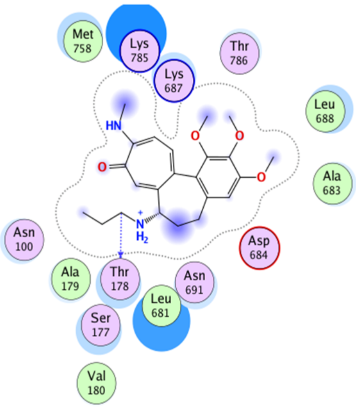	-4.9	Asn100<br>**Thr178**<br>**Leu681**<br>Lys687<br>Leu688<br>**Asn691**<br>**Lys785**
6	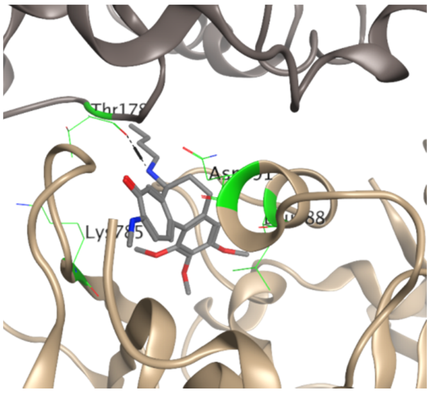	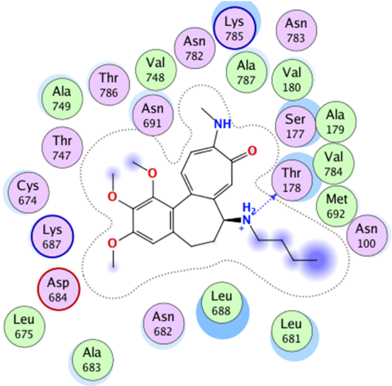	-19.4	**Thr178**<br>**Leu688**<br>Asn691<br>Lys785
7	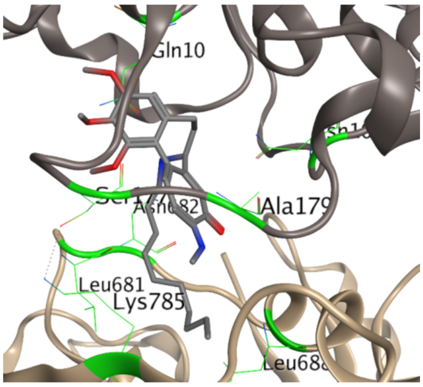	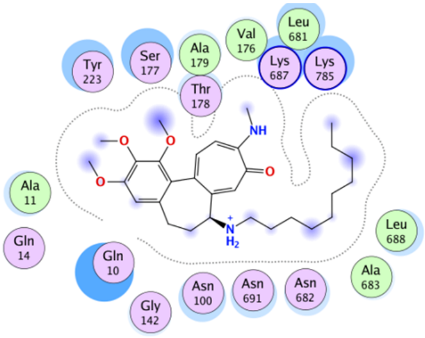	-16.9	**Gln10**<br>**Asn100**<br>**Ser177**<br>Ala179<br>Leu681<br>**Asn682**<br>Leu688<br>Asn691<br>**Lys785**

8	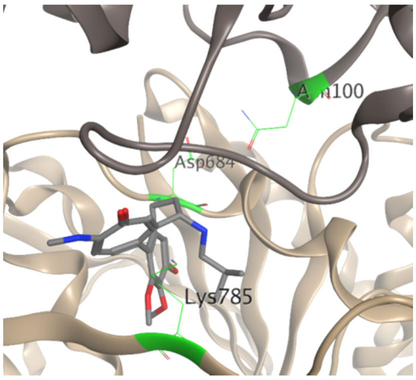	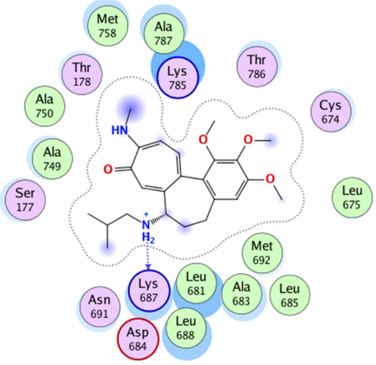	-1.0	Asn100<br>Asp684<br>**Leu688**<br>**Lys785**
9	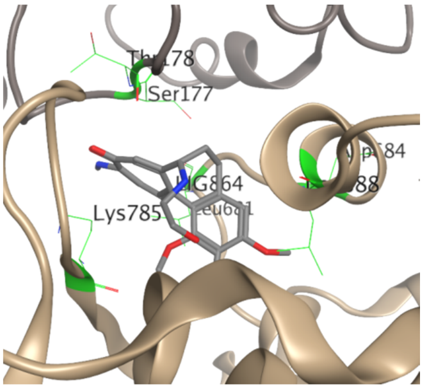	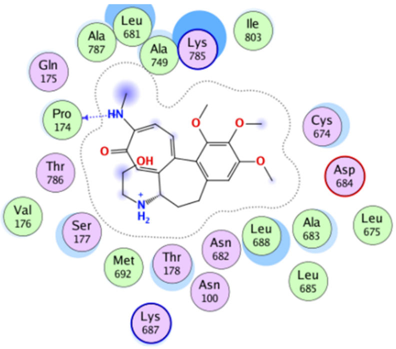	-2.6	**Ser177**<br>**Thr178**<br>Leu681<br>Asp684<br>**Leu688**<br>**Lys785**
10	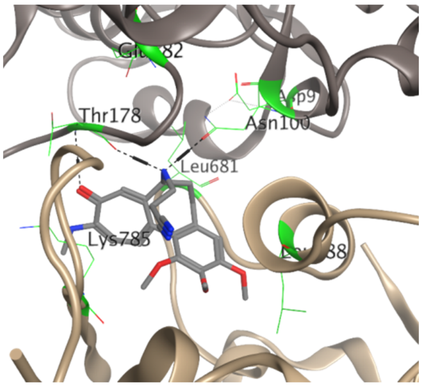	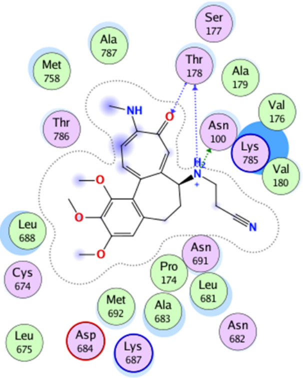	5.0	Asp97<br>**Asn100**<br>**Thr178**<br>Glu182<br>Leu681<br>**Leu688**<br>**Lys785**
11	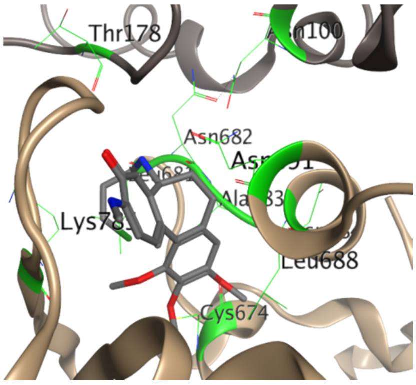	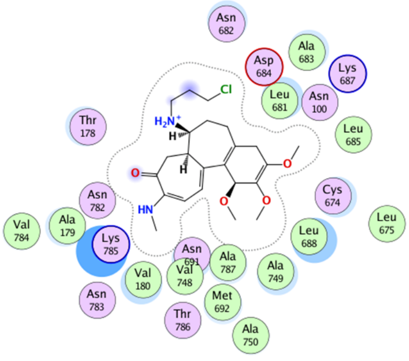	-19.9	Asn100<br>**Thr178**<br>Cys674<br>**Leu681**<br>**Asn682**<br>Ala683<br>Asp684<br>**Leu688**<br>Asn691<br>Lys785

**12**	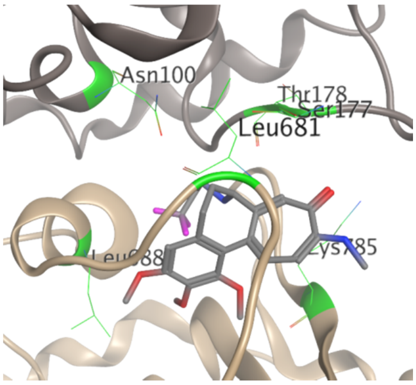	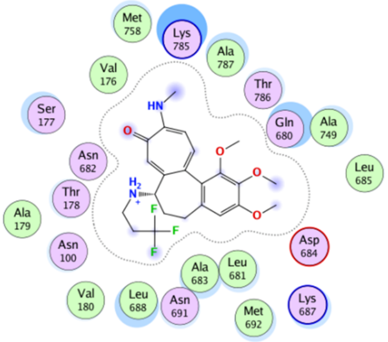	8.7	**Asn100**<br>Ser177<br>Thr178<br>Leu681<br>Leu688<br>**Lys785**
**13**	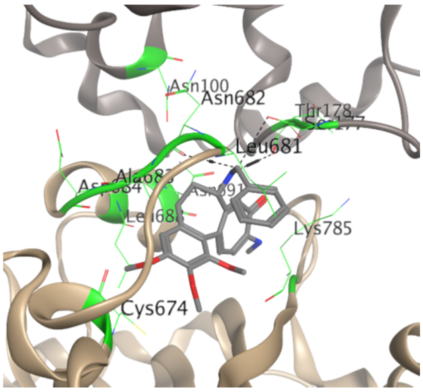	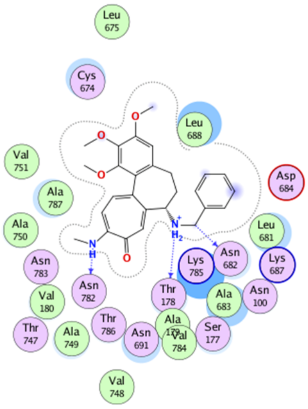	-21.4	Asn100<br>Ser177<br>**Thr178**<br>Cys674<br>**Leu681**<br>**Asn682**<br>Ala683<br>Asp684<br>**Leu688**<br>Asn691<br>**Lys785**
**14**	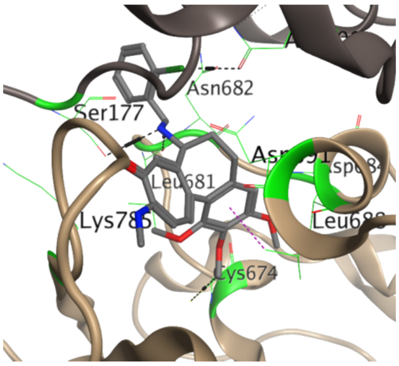	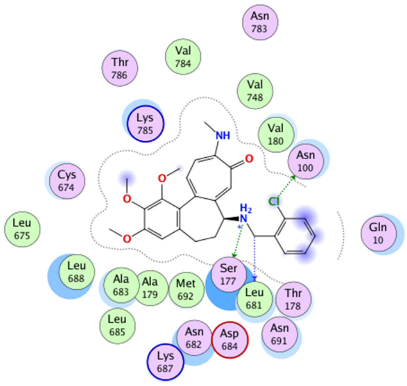	-31.5	**Asn100**<br>**Ser177**<br>Cys674<br>**Leu681**<br>**Asn682**<br>**Asp684**<br>**Leu688**<br>Lys785
**15**	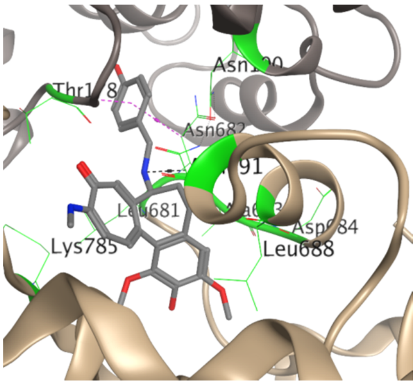	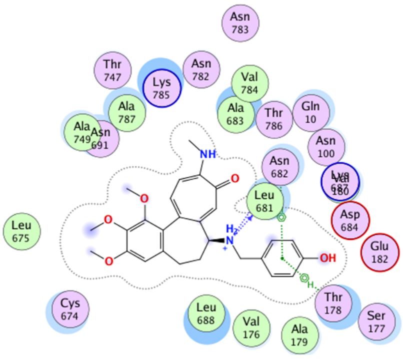	-27.1	**Asn100**<br>**Thr178**<br>**Leu681**<br>**Asn682**<br>Ala683<br>**Asp684**<br>**Leu688**<br>Asn691<br>Lys785

**16**	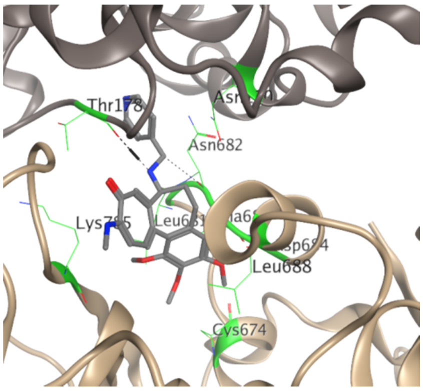	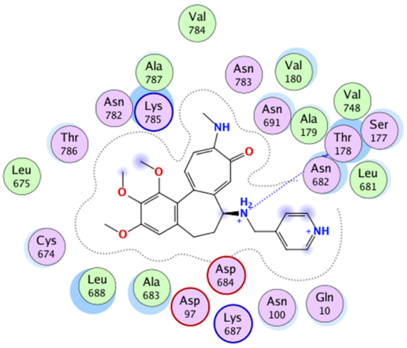	-27.4	**Asn100**<br>**Thr178**<br>Cys674<br>Leu681<br>**Asn682**<br>Ala683<br>**Asp684**<br>**Leu688**<br>Lys785
**17**	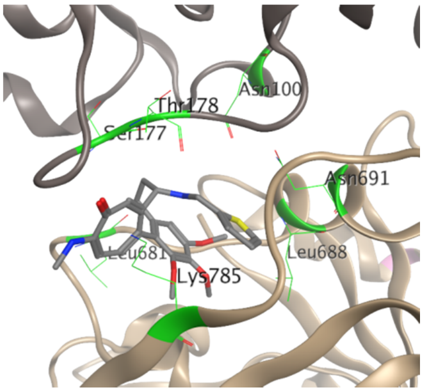	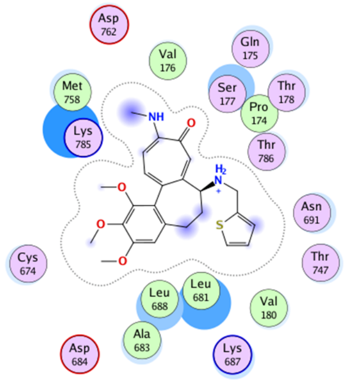	-3.6	Asn100<br>Ser177<br>**Thr178**<br>**Leu681**<br>Leu688<br>Asn691<br>**Lys785**

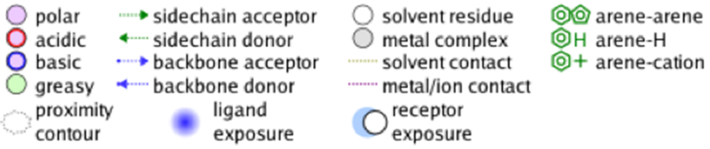

In 3D illustrations the interacting residues predicted from pairwise per-residue binding free energy decomposition calculations (E < -2 kcal/mol) are shown in stick representation and their carbons and the ribbon are colored as green. Tubulin is shown in cartoon representation. Hydrogen bonds and their directionality are represented as black dashed arrows. The structures are color coded as follows: tubulin αI, brown; tubulin βI, beige. Ligands are displayed with stick and the atoms are colored as O (red), C (gray), N (blue), S (yellow), Cl (green) and F (pink). Binding energy defines the affinity of binding of colchicine derivatives complexed with tubulin βI. Binding energies are predicted by the MM/GBSA method. The last column contains information about active residues with binding free energy decomposition (E < -2 kcal/mol) and the residues with (E < -3 kcal/mol) are highlighted in bold. The last line contains the graphical key to help interpret the 2D part of the ligand interactions panel.

MATERIALS AND METHODS

General

All solvents, substrates and reagents were obtained from TriMen Chemicals (Poland) or Sigma Aldrich and were used without further purification. Spectral grade solvents were stored over 3 Å molecular sieves for several days. TLC analysis was performed using pre-coated glass plates (0.2 mm thickness, GF-254, pore size 60 Å) from Agela Technologies and spots were visualized by UV-light. Products were purified by flash chromatography using high-purity grade silica gel (pore size 60 Å, 230 - 400 mesh particle size, 40-63 μm particle size) from SiliCycle Inc. Solvents were removed using a rotary evaporator.

Spectroscopic measurements

NMR spectra were recorded on Bruker Avance DRX 500 (^{1}H NMR at 500 MHz and ^{13}C NMR at 126 MHz) magnetic resonance spectrometers. ^{1}H NMR spectra are reported in chemical shifts downfield from TMS using the respective residual solvent peak as internal standard ($CDCl_3$ δ 7.26 ppm, CD_2Cl_2 δ 5.32 ppm and CD_3CN δ 1.94). ^{1}H NMR spectra are reported as follows: chemical shift (δ, ppm), multiplicity (s = singlet, d = doublet, t = triplet, q = quartet, dd = doublet of doublets, dt = doublet of triplets, dq = doublet of quartets, m = multiplet), coupling constant (J) in Hz, and integration. ^{13}C NMR spectra are reported in chemical shifts downfield from TMS using the respective residual solvent peak as internal standard ($CDCl_3$ δ 77.2 ppm, CD_2Cl_2 δ 53.8 ppm, CD_3CN δ 1.3 and 118.3).

Electrospray ionization (ESI) mass spectra were obtained on a Waters Alliance 2695 separation module with a PDA 2996 UV detector and Waters Micromass ZQ 2000 mass detector equipped with Restek Ultra Biphenyl 50 x 3 mm, 3 μm column eluted with 0.3 mL/min flow of 3-100% gradient (over 6 min) of acetonitrile in water.

Synthesis of 2 and 3

Synthesis of 10-*N*-methylaminocolchicine **2** and *N*-deacetyl-10-methylamino-10-demethoxycolchicine **3** was performed according to the previously published procedure [1].

Compound **2**

To a solution of **1** (1.0 equiv.) in EtOH a methylamine (solution 33% in EtOH, 10.0 equiv.) was added. The mixture was stirred at reflux for 24 h and then concentrated under reduced pressure to dryness. The residue was purified using column flash chromatography (silica gel; DCM/MeOH, 40/1 *v/v*) and next lyophilized from dioxane to give the pure product **2** as a yellow solid with a yield of 80%.

ESI-MS for $C_{22}H_{26}N_2O_5$ (m/z): $[M+H]^+$ 399, $[M+Na]^+$ 421, $[2M+H]^+$ 797, $[2M+Na]^+$ 819, $[M-H]^-$ 397, $[M+HCOO^-]^-$ 443.

^{1}H NMR (500 MHz, $CDCl_3$) δ 8.70 (d, J = 6.4 Hz, 1H), 7.58 (s, 1H), 7.46 (d, J = 11.1 Hz, 1H), 7.28 – 7.25 (m, 1H), 6.58 (d, J = 11.3 Hz, 1H), 6.52 (s, 1H), 4.73 – 4.64 (m, 1H), 3.93 (s, 3H), 3.88 (s, 3H), 3.61 (s, 3H), 3.08 (d, J = 5.4 Hz, 3H), 2.47 – 2.43 (m, 1H), 2.37 – 2.31 (m, 1H), 2.29 – 2.22 (m, 1H), 2.02 – 1.96 (m, 1H), 1.94 (s, 3H).

^{13}C NMR (126 MHz, $CDCl_3$) δ 175.1, 170.2, 155.2, 152.9, 151.6, 151.1, 141.5, 139.4, 134.7, 130.4, 126.9, 122.8, 108.3, 107.2, 61.5, 61.4, 56.2, 52.7, 37.1, 30.2, 29.5, 22.7.

Compound **3**

To a solution of compound **2** (1.0 equiv.) in dioxane, 2M HCl (10.0 equiv.) was added and the mixture was stirred at reflux. Reaction progress was monitored by LC-MS. Then the reaction mixture was

neutralized with 4M NaOH to pH~10 and extracted four times with EtOAc. The organic layers were combined, washed with brine, dried over Na_2SO_4, filtered off and evaporated under reduced pressure. The residue was purified using column flash chromatography (silica gel; DCM/MeOH, 20/1 *v/v*) and next lyophilized from dioxane to give the pure product **3** as a yellow solid with a yield of 73%.

ESI-MS for $C_{20}H_{24}N_2O_4$ (m/z): $[M+H]^+$ 357, $[M+Na]^+$ 379, $[2M+Na]^+$ 735.

^{1}H NMR (500 MHz, $CDCl_3$) δ 7.61 (s, 1H), 7.33 (d, J = 11.1 Hz, 1H), 7.23 – 7.21 (m, 1H), 6.50 (s, 1H), 6.50 (d, J = 11.4 Hz, 2H), 3.87 (s, 3H), 3.87 (s, 3H), 3.75 – 3.72 (m, 1H), 3.59 (s, 3H), 3.05 (d, J = 5.5 Hz, 3H), 2.41 – 2.37 (m, 1H), 2.33 – 2.31 (m, 2H), 2.25 (s, 2H), 1.71 – 1.61 (m, 1H).

^{13}C NMR (126 MHz, $CDCl_3$) δ 175.6, 155.0, 153.3, 152.8, 150.6, 141.1, 138.7, 135.4, 129.7, 126.6, 123.7, 107.4, 106.9, 61.2, 60.8, 56.1, 54.0, 40.9, 30.7, 29.5.

In vitro **antiproliferative activity**

Cell lines and culturing conditions

Four human cancer cell lines and one murine normal cell line were used to evaluate antiproliferative activity of colchicine and its derivatives **1-17**: human lung adenocarcinoma (A549), human breast adenocarcinoma (MCF-7), human colon adenocarcinoma cell lines sensitive and resistant to doxorubicin (LoVo) and (LoVo/DX) respectively, and normal murine embryonic fibroblast cell line (BALB/3T3). The A549 cell line was purchased from the European Collection of Authenticated Cell Cultures (ECACC, Salisbury, UK). The MCF-7, LoVo and LoVo/DX cell lines was purchased from American Type Culture Collection (ATCC, Manassas, VA, USA). All the cell lines are maintained in the Institute of Immunology and Experimental Therapy (IIET), Wroclaw, Poland.

Human lung adenocarcinoma cell line was cultured in a mixture of OptiMEM and RPMI 1640 (1:1) medium (IIET, Wroclaw, Poland), supplemented with 5% fetal bovine serum HyClone (GE Healthcare, USA) and 2 mM L-glutamine (Sigma-Aldrich, Germany). Human breast adenocarcinoma cell line was cultured in mixture of Eagle medium (IIET, Wroclaw, Poland), supplemented with 10% fetal bovine serum, 2 mM L-glutamine, 8 μg/mL insulin and 1% amino acids (Sigma-Aldrich, Germany). Human colon adenocarcinoma cell lines were cultured in mixture of OptiMEM and RPMI 1640 (1:1) medium (IIET, Wroclaw, Poland), supplemented with 5% fetal bovine serum HyClone (GE Healthcare, USA), 2 mM L-glutamine, 1 mM sodium pyruvate (Sigma-Aldrich, Germany) and 10 μg/100 mL doxorubicin (Accord) for LoVo/DX. Murine embryonic fibroblast cells were cultured in Dulbecco medium (Gibco), supplemented with 10% fetal bovine serum (GE Healthcare, USA) and 2 mM L-glutamine (Sigma-Aldrich, Germany). All culture media contained antibiotics: 100 U/mL penicillin (Polfa-Tarchomin, Poland) and 0,1 mg/mL streptomycin (Sigma Aldrich, Germany). All cell lines were cultured during entire experiment in humid atmosphere at 37°C and 5% CO_2.

Cell viability assays

Twenty-four hours before adding the tested compounds, all cell lines were seeded in 384-well plates (Sarstedt, Germany) in appropriate media with $1.0x10^3$ cells per well for A549 cell line, $1.5x10^3$ cells per well for MCF-7 cell line and $2.0x10^3$ cells per well for LoVo, LoVo/DX and BALB/3T3 cell lines. All cell lines were exposed to each tested agent at different concentrations in the range 100–0.001 μg/mL for 72 h. The cells were also exposed to the reference drug cisplatin (Teva Pharmaceuticals, Poland) and doxorubicin (Accord Healthcare Limited, UK). Additionally, all cell lines were exposed to DMSO (solvent used for tested compounds) (POCh, Poland) at concentrations corresponding to those present in tested agents dilutions. After 72 h sulforhodamine B assay (SRB) was performed [2].

SRB

After 72 h of incubation with the tested compounds, the cells were fixed in situ by gently adding of 30 µL per well of cold 50% trichloroacetic acid TCA (POCh, Poland) and were incubated at room temperature for one hour. Then the wells were washed four times with water and air dried. Next, 25 µL of 0.1% solution of sulforhodamine B (Sigma-Aldrich, Germany) in 1% acetic acid (POCh, Poland) were added to each well and plates were incubated at room temperature for 0.5 h. After incubation time, unbound dye was removed by washing plates four times with 1% acetic acid, whereas stain bound to cells was solubilized with 70µl of 10 mM Tris base (Sigma-Aldrich, Germany). Absorbance of each solution was read off from a Synergy H4 Hybrid Multi-Mode Microplate Reader (BioTek Instruments, USA) at the 540 nm wavelength.

Results are presented as mean IC_{50} (concentration of the tested compound, that inhibits cell proliferation by 50%) ± standard deviation. IC_{50} values were calculated in Cheburator 0.4, Dmitry Nevozhay software (version 1.2.0 software by Dmitry Nevozhay, 2004–2014, http://www.cheburator.nevozhay.com, freely available) for each experiment [3]. Compounds at each concentration were tested in triplicates in single experiment and each experiment was repeated at least three times independently.

Molecular docking studies

Ligand preparation

The ligand structures were prepared using Ligprep from the Schrödinger suite [4]. Conformations and tautomeric states were assigned to the ligands by following the ligand preparation protocol implemented in Schrödinger suite with default settings. LigPrep generates variants of the same ligand with different tautomeric, stereochemical, and ionization properties.

Tubulin model

The tubulin crystal structures available in the PDB are those for bovine protein. The bovine tubulin structure of tubulin (PDB ID: 1SA0) [5] was used as a template to construct the homology model of human αβ-tubulin isotypes (βI (UniProtKb: P07437), which is the most abundant isotype in most tumors using the Molecular Operating Environment (MOE) software package [6]. The sequence corresponding to the gene TUBA1A (UniProt ID: Q71U36) was chosen as a reference sequence for human tubulin, whereas the gene TUBB associated to βI isoform (UniProt ID: P07437) was chosen for human tubulin. Homology modeling was performed using MOE by setting the number of generated models to 10 and by selecting the final model based on MOE's generalized Born/volume integral (GB/VI) scoring function.

Molecular dynamics simulations

The missing hydrogens for heavy atoms were added using the tLEAP module of AMBER 14 with the AMBER14SB force field [7]. The protonation states of all ionizable residues were determined at pH = 7 using the MOE program [6]. Each protein model was solvated in a 12 Å box of TIP3P water. In order to bring the salt concentration to the physiological value of 0.15 M, 93 Na^+ ions and 57 Cl^- ions were added. Minimization of the structure was carried out in two steps, using the steepest descent and conjugate gradient methods successively. At first, minimization was made in 2 ps on solvent atoms only, by restraining the protein-ligand complex. Next, minimization was run without the restraint in 10 ps. After minimization, the molecular dynamics (MD) simulations were carried out in three steps: heating, density equilibration, and production. At first, each solvated system was heated to 298 K for 50 ps, with weak restraints on all backbone atoms. Next, density equilibration was carried out for 50 ps of constant pressure equilibration at

298 K, with weak restraints. Finally, MD production runs were performed for all systems for 70 ns. The root-mean-square deviation (RMSD) of both the entire tubulin structure and the colchicine-binding site were found to reach a plateau after 40 ns. Clustering analysis of the last 30 ns of the generated MD trajectory was carried out using the Amber's CPPTRAJ program [8] to identify representative conformations of the tubulin dimer. Clustering was made via the hierarchical agglomerative approach using the RMSD of atoms in the colchicine-binding site as a metric. An RMSD cutoff of 1.0 Å was set to differentiate the clusters. On the basis of the clustering analysis, three representative structures of the tubulin dimer were found. The docking was performed on all the three representative structures and the one with the highest docking score was selected, which was the largest cluster (about 70% of the simulation) conformation of the tubulin structure. During the modeling, the cofactors including GTP, GDP, colchicine, and the magnesium ion located at the interface between α - and β-monomers were kept as part of the environment and included in the refinement step.

Docking simulations

We used the AutoDock Vina [9] and DOCK (http://dock.compbio.ucsf.edu/) programs to predict the binding pose of the ligands under flexible ligand and rigid receptor conditions. Dockbox package was used to facilitate preparation of docking inputs and post-processing of the docking results [10]. Docking simulations performed with a cubic box (size 30.0 Å) were centered at the middle of binding pockets and the docking was run separately on the tubulin structure. Every generated pose was energy-minimized using Amber14 by keeping the protein fixed and was re-scored using the MOE's GBVI/WSA dG scoring function [6]. No constraints were applied in the docking studies. For each compound/protein-structure pair, the pose with the best score was identified and used as an initial configuration for molecular mechanics Gibbs–Boltzmann surface area MM/GBSA computations.

Binding energy calculations using MM/GBSA method

The MM/GBSA technique is used to calculate the free energy associated with the binding of double modified colchicine derivatives [11]. This method combines molecular mechanics with continuum solvation models. We performed MM-GBSA integrated in Amber. The binding free energy is estimated as:

$$\Delta G_{bind} = \bar{G}_{complex} - [\bar{G}_{protein} + \bar{G}_{ligand}] \quad (1)$$

where G is the average free energy of the complex, protein, and ligand, are calculated according to the equation:

$$\bar{G} = \bar{E}_{M+} \bar{G}_{solvation} - T\bar{S} \quad (2)$$

where EMM are determined with the SANDER program and represent the internal energy (bond, angle, and dihedral), van der Waals and electrostatic interactions (see equation (3)). TS is the entropy contribution estimated using normal mode (nmode) analysis.

$$\bar{\mathrm{E}}_{\mathrm{M}} = \bar{\mathrm{E}}_{\mathrm{int}} + \bar{\mathrm{E}}_{\mathrm{elec}} + \bar{\mathrm{E}}_{\mathrm{vdW}} \quad (3)$$

The solvation free energy can be calculated as the sum of polar and nonpolar contributions. The polar parts are obtained by using the generalized-born (GB) model—resulting in the MM/GBSA method, whereas the

nonpolar terms are estimated from a linear relation (equation 4) to the solvent accessible surface area (SASA).

$$\bar{G}_{non-polar} = \gamma\, SASA + b \quad (4)$$

In the present study, a 2 ns-duration MD trajectory was run in TIP3P water using Amber14, for every top pose generated at the end of the docking step. It is worth noting that to assess the performance of MM/GBSA methodology [12], we evaluated the prediction accuracy of this method by various simulation protocols including 1 ns MD production calculations using PDBbind data set. Too long an MD simulation could be prejudicious for the overall success of the MM/GBSA method. According to this study and the common practice to calculate binding energies using MM/GBSA, we have decided to run MD production simulation for 2 ns. The MM/GBSA calculations were performed on a subset of 200 frames collected at regular time intervals from the trajectory. For PB calculations, an ionic strength of 0.0 nM (istrng = 0.0) and a solvent probe radius of 1.6 Å (prbrad = 1.6) were used. For GB calculations, the igb parameter was set to 5, which corresponds to a modified GB model equivalent to model II in reference.

Free energy decomposition analysis

The interaction between inhibitors and each residue were computed using the MM/GBSA decomposition process by the mm_gbsa program in AMBER 12.0. The binding interaction of each inhibitor-residue pair includes three energy terms: van der Waals contribution (ΔE_{vdw}), electrostatic contribution (ΔE_{ele}), and solvation contribution (ΔGGB + ΔGSA), in which ΔE_{vdw} and ΔE_{ele} are van der Waals and electrostatic interactions between the inhibitor and each protein residue that could be computed by the Sander program in AMBER 12.0. The polar contribution of desolvation (ΔGGB) was calculated using the generalized Born (GB) model. The nonpolar contribution of desolvation (ΔGSA) was computed based on SASA determined with the ICOSA method. All energy components were calculated using 300 snapshots extracted from the MD trajectory from 7 to 10 ns (Molecular Dynamics Simulation of Tryptophan Hydroxylase-1: Binding Modes and Free Energy Analysis to Phenylalanine Derivative Inhibitors paper).

LC-MS chromatograms and mass spectra, ^{1}H NMR and ^{13}C NMR spectra of compounds 2-17.

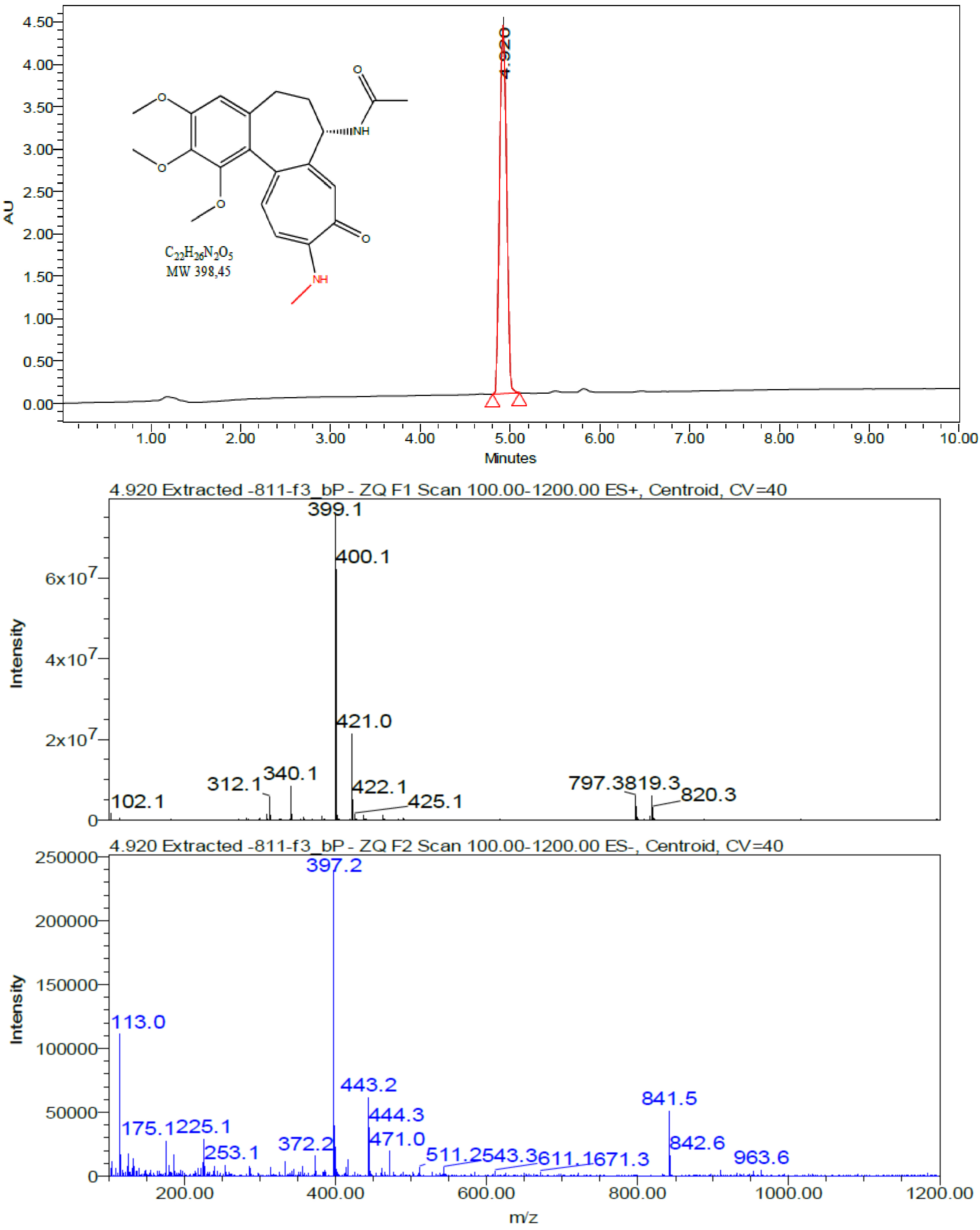

Figure S1. The LC-MS chromatogram and mass spectra of **2**.

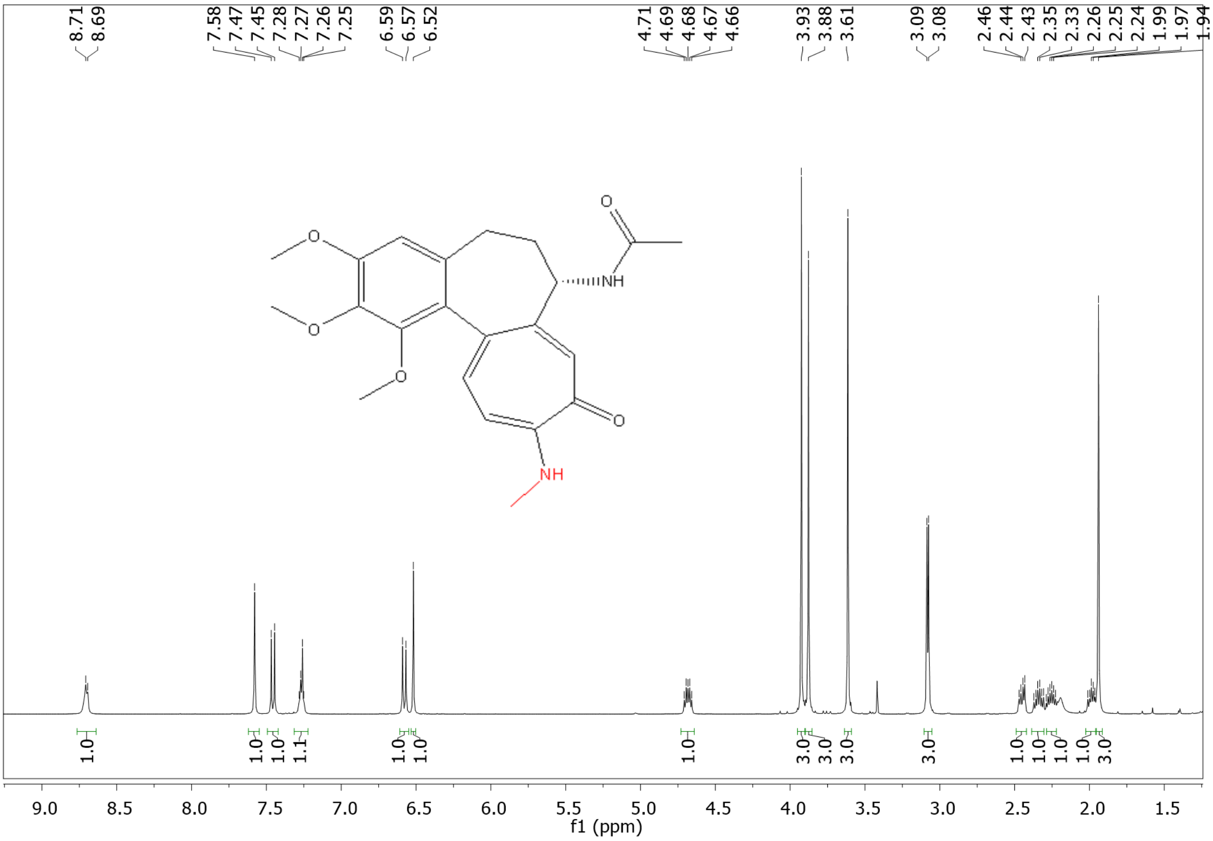

Figure S2. The ^{1}H NMR spectrum of **2** in $CDCl_3$.

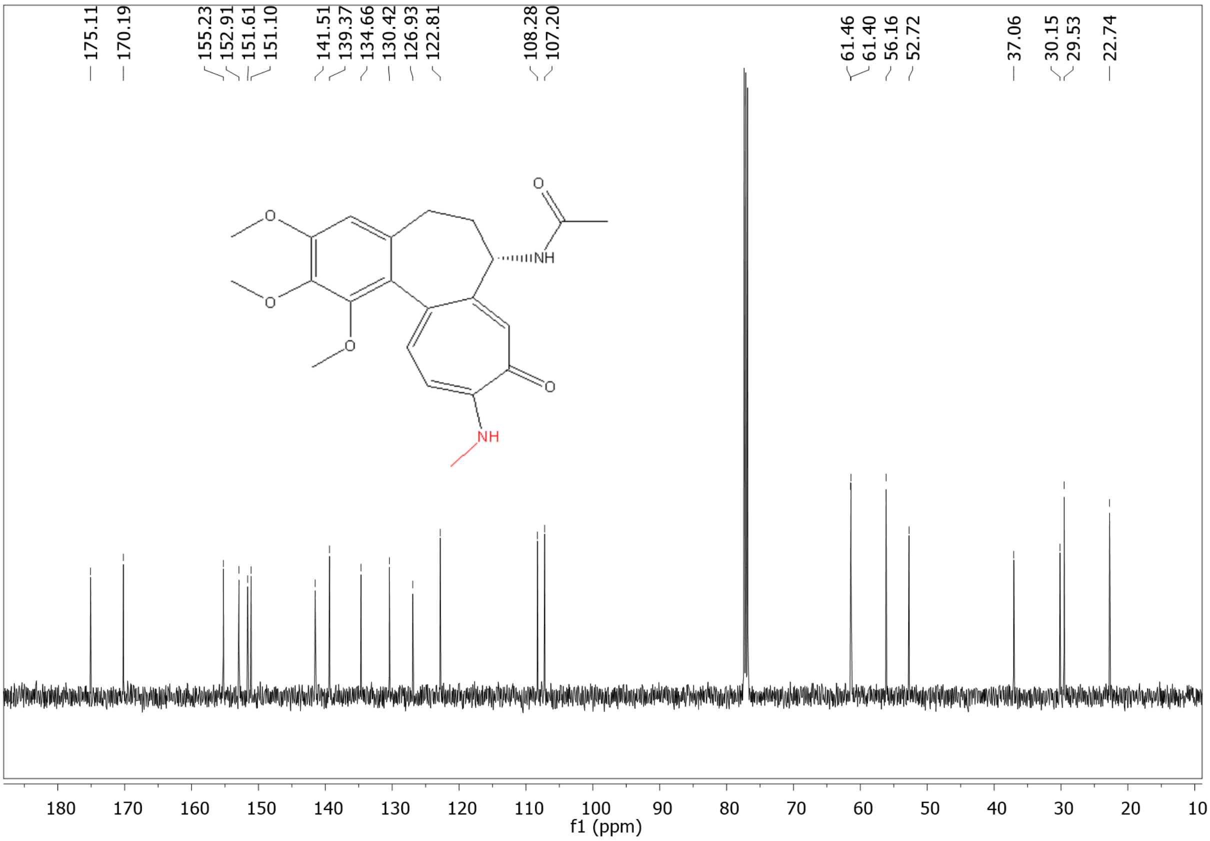

Figure S3. The ^{13}C NMR spectrum of **2** in $CDCl_3$.

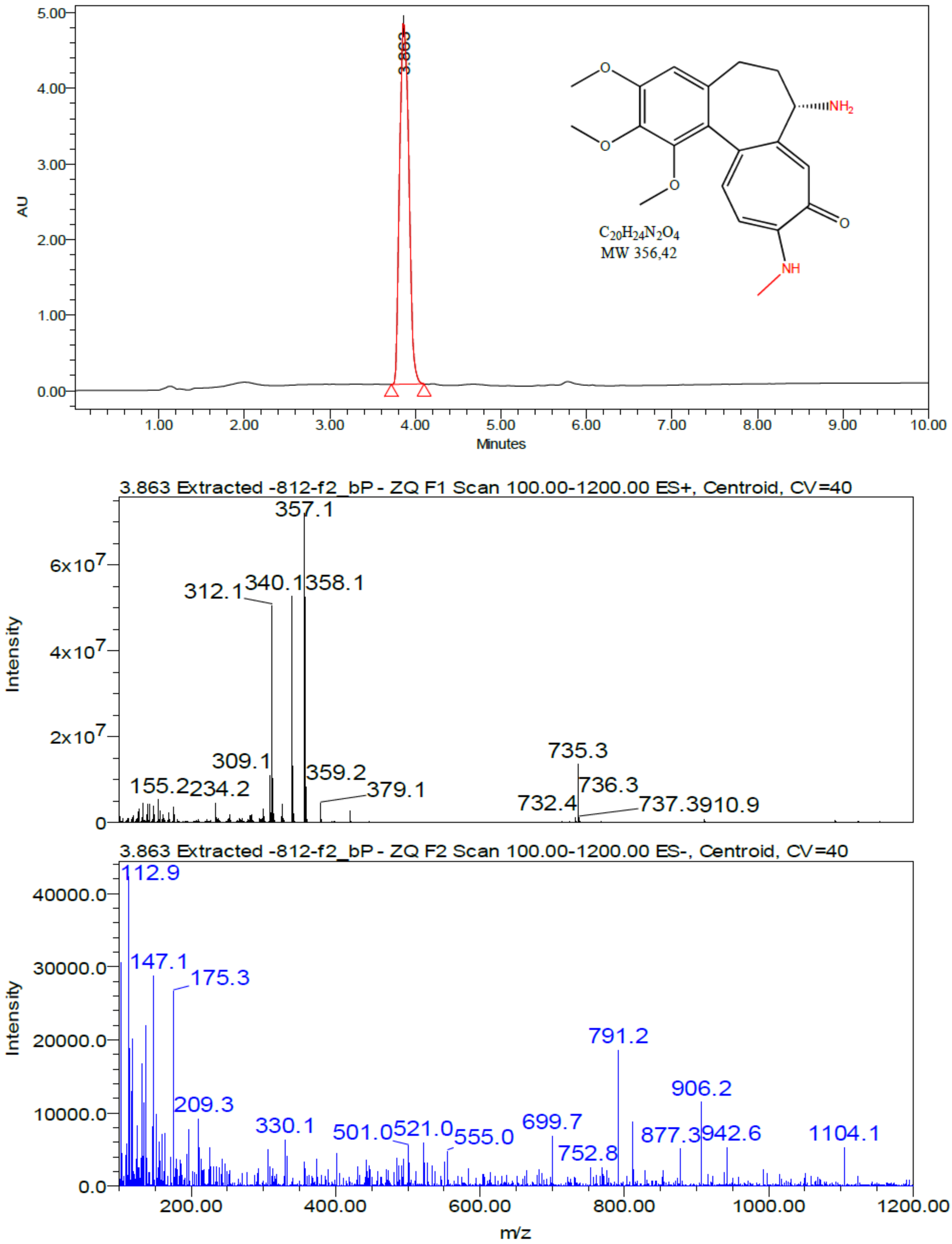

Figure S4. The LC-MS chromatogram and mass spectra of **3**.

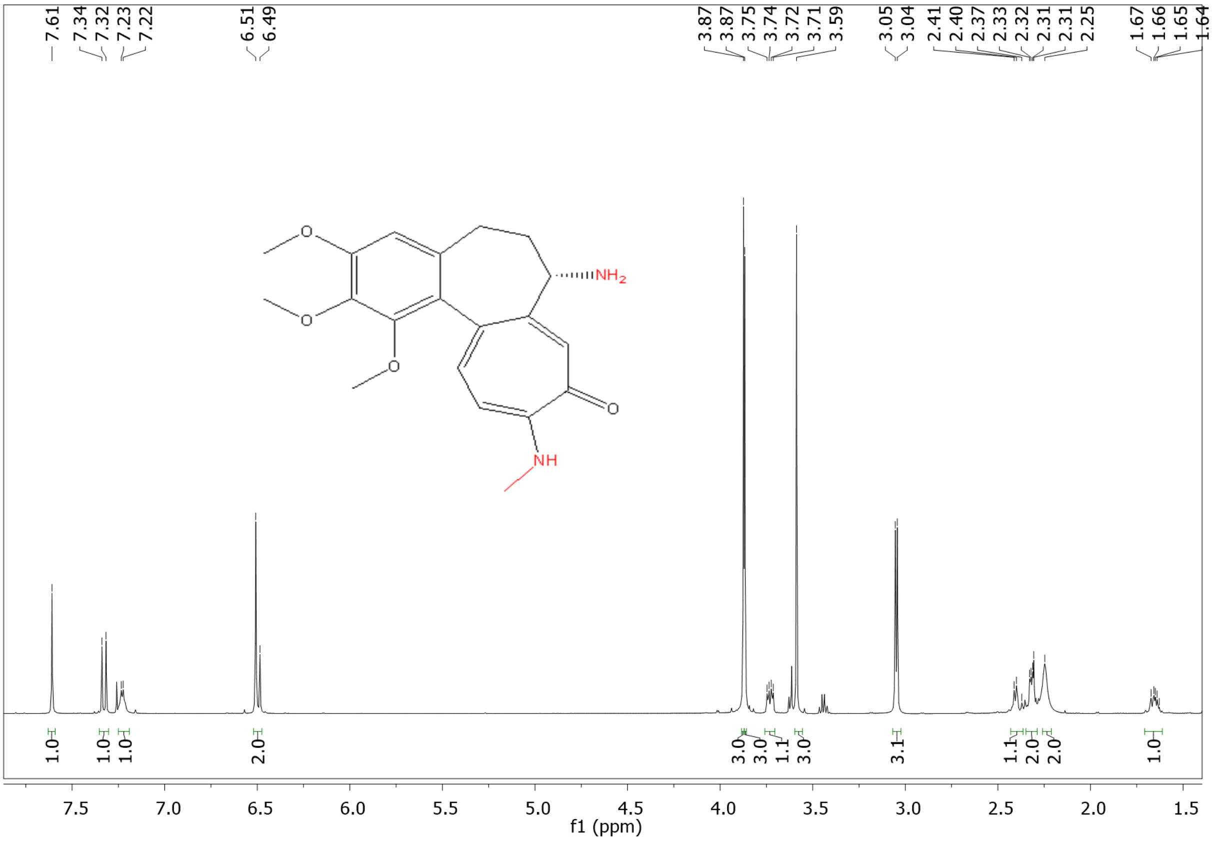

Figure S5. The ^{1}H NMR spectrum of **3** in $CDCl_3$.

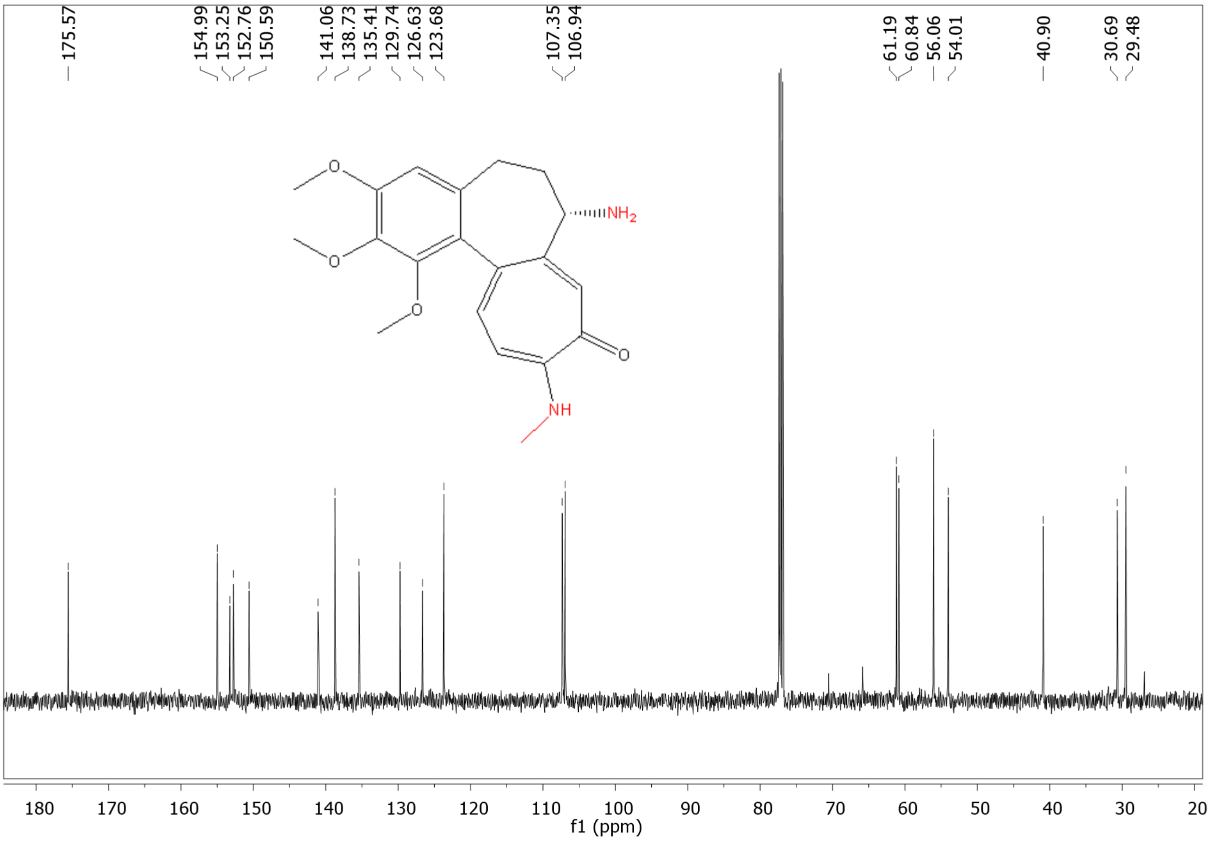

Figure S6. The ^{13}C NMR spectrum of **3** in $CDCl_3$.

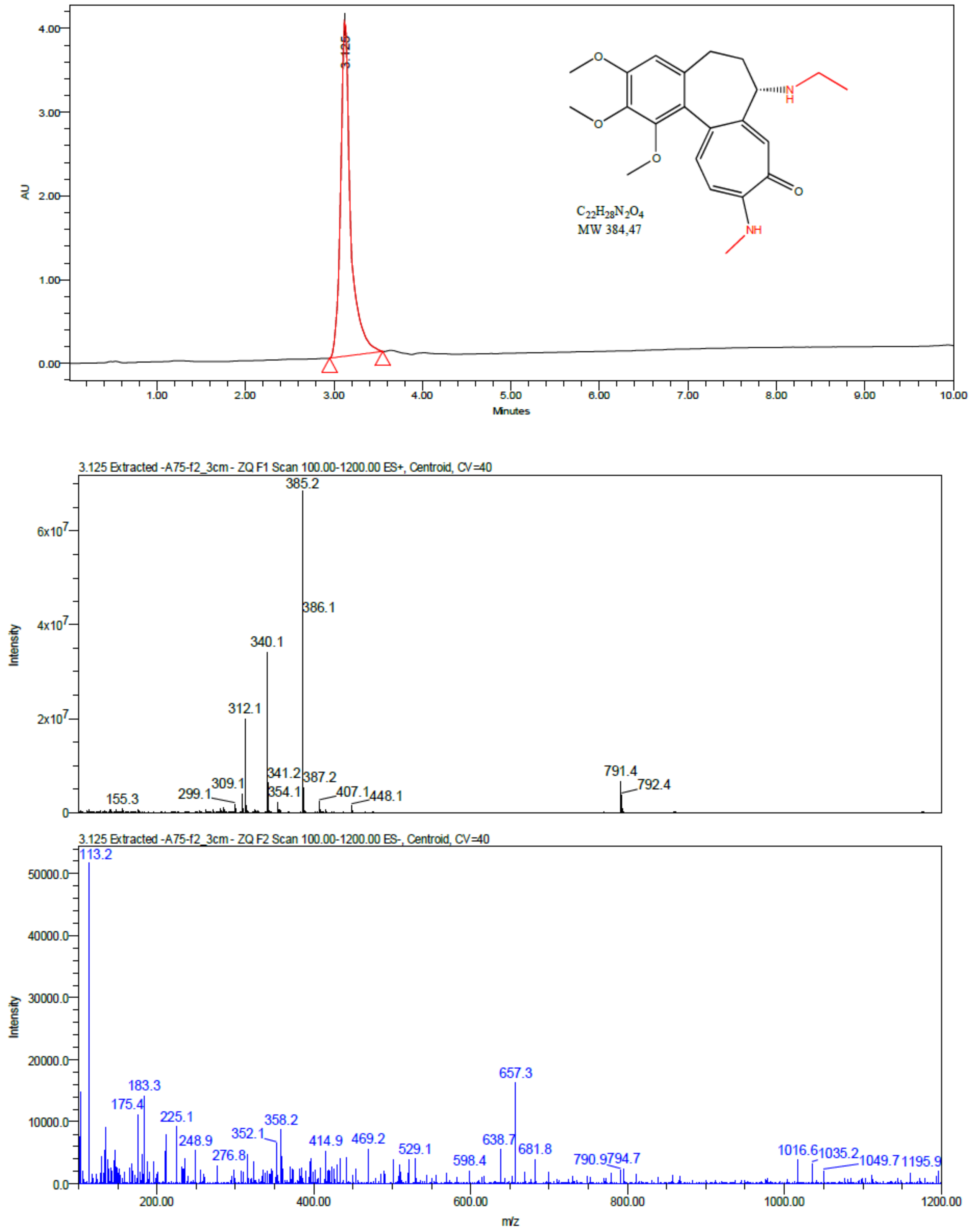

Figure S7. The LC-MS chromatogram and mass spectra of **4**.

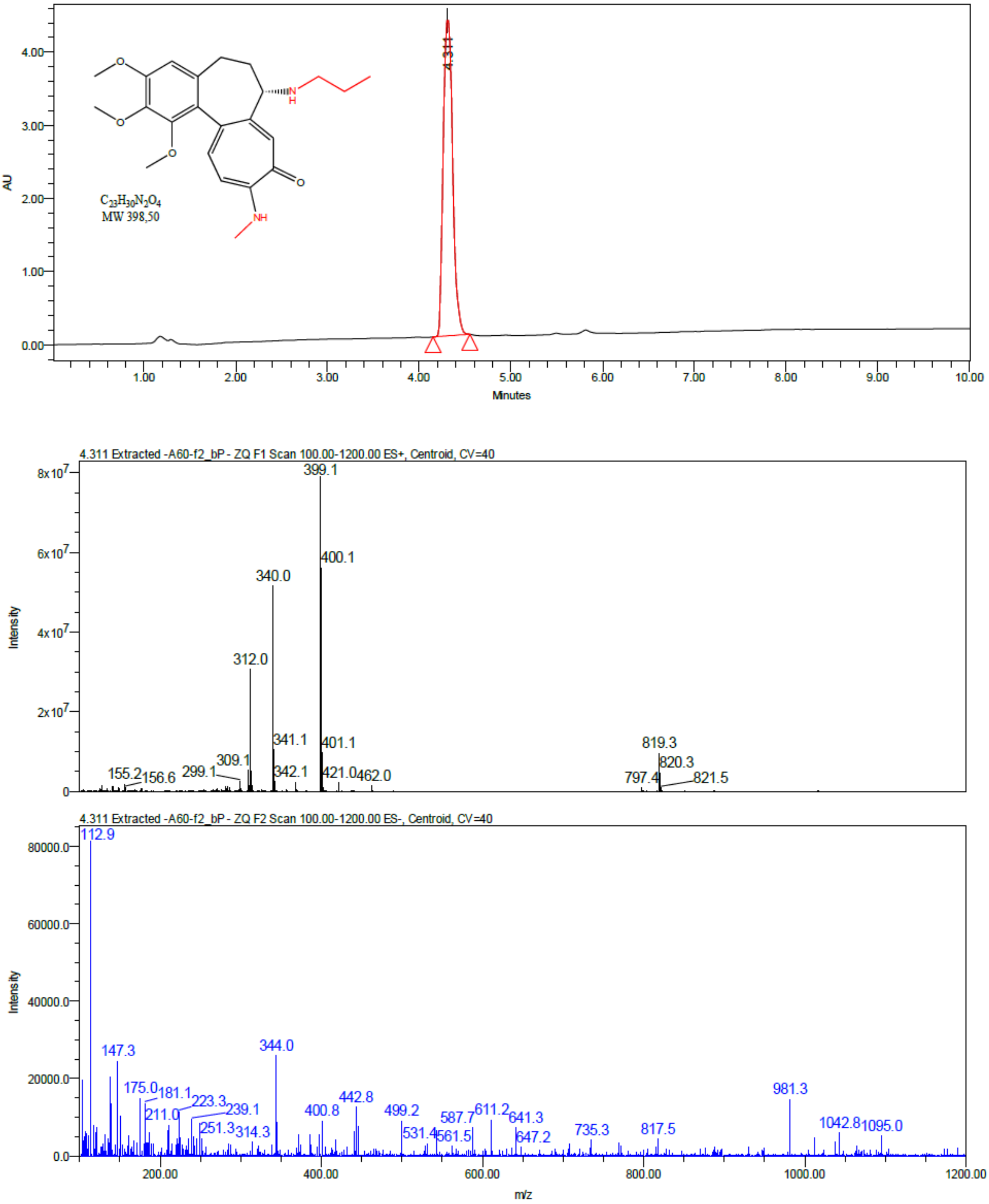

Figure S8. The LC-MS chromatogram and mass spectra of **5**.

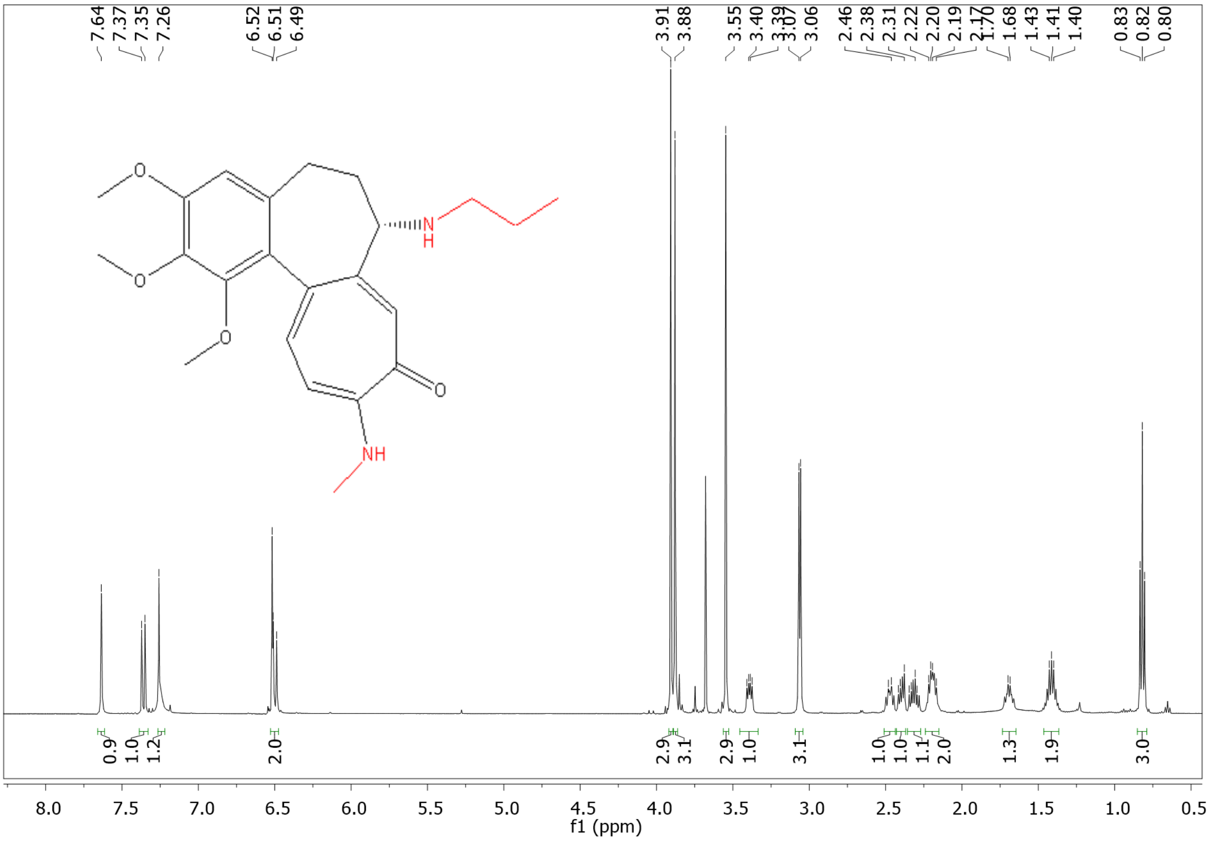

Figure S9. The ^{1}H NMR spectrum of **5** in $CDCl_3$.

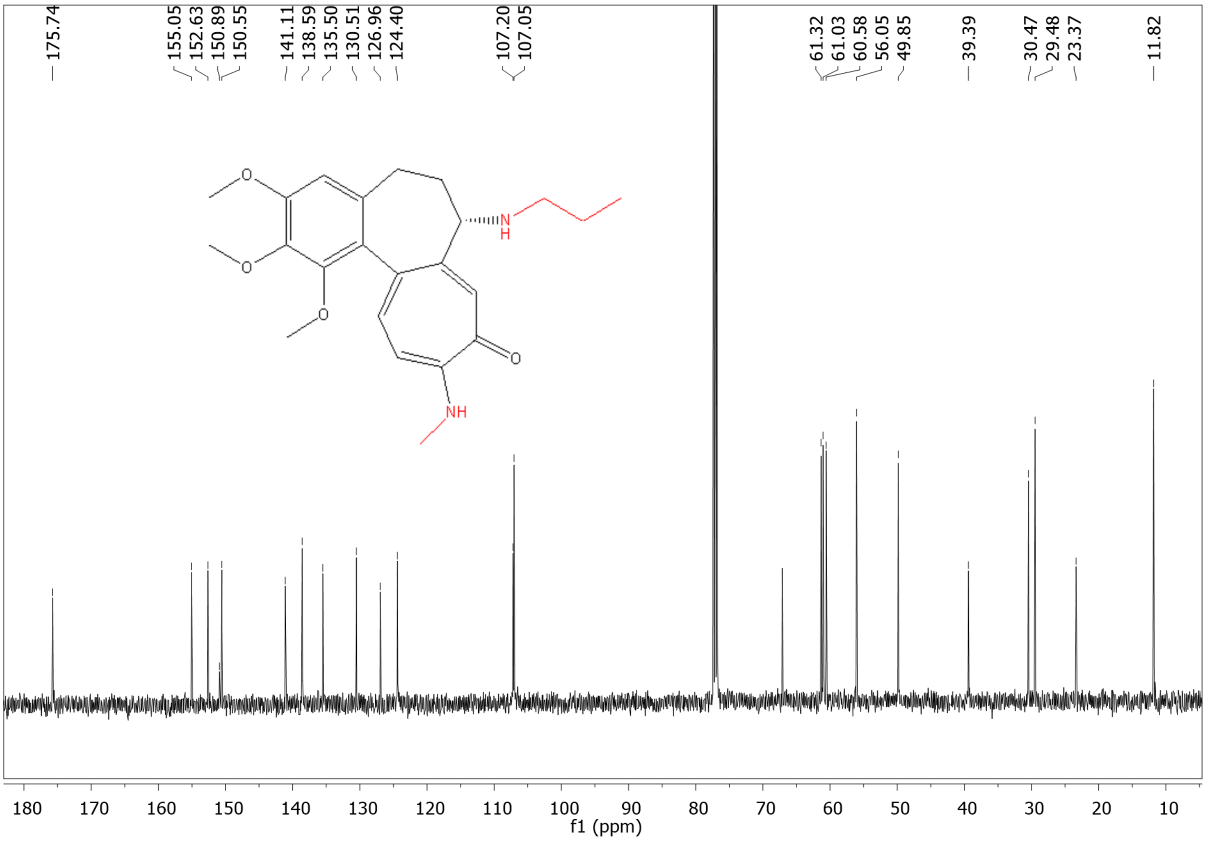

Figure S10 The ^{13}C NMR spectrum of **5** in $CDCl_3$.

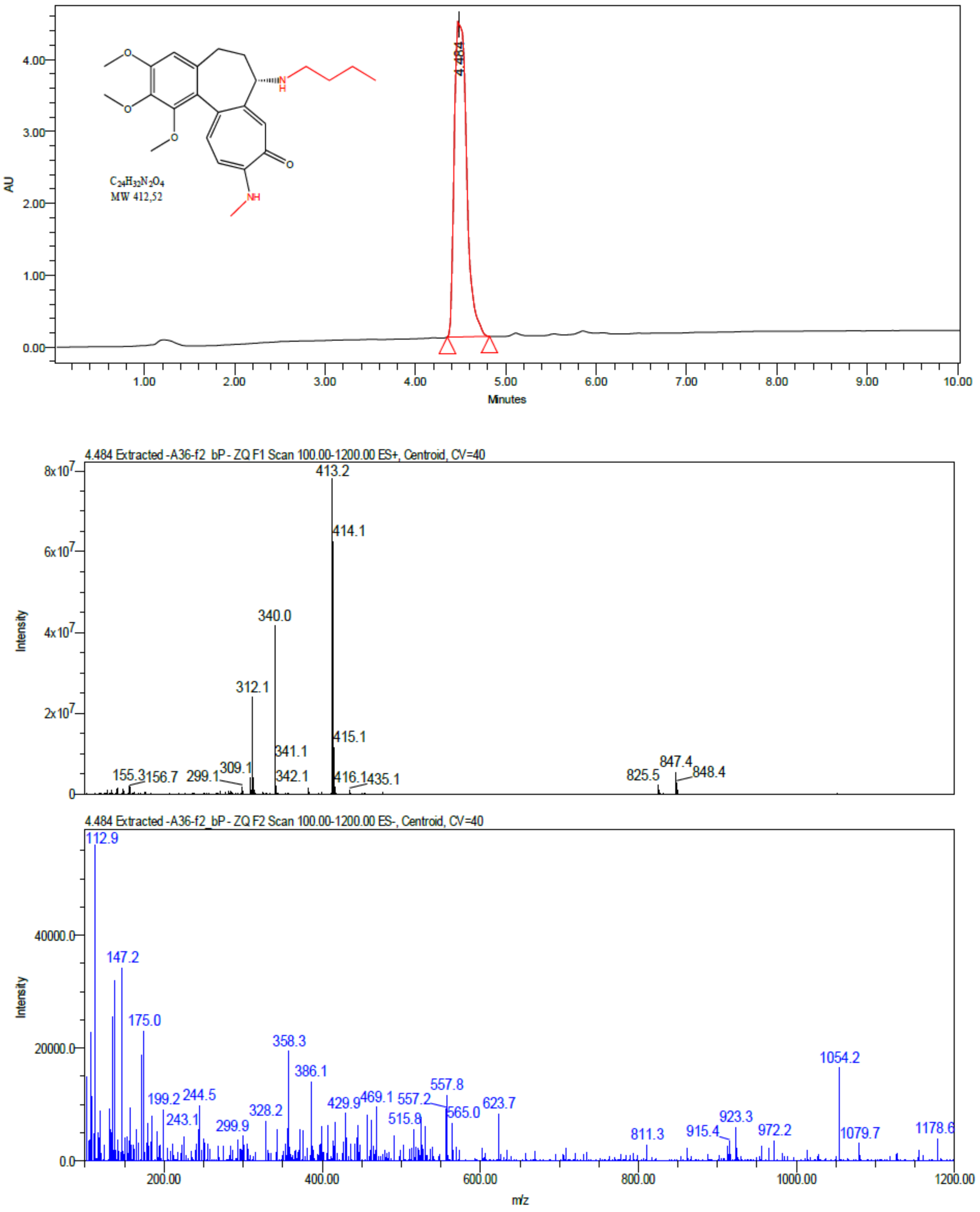

Figure S11. The LC-MS chromatogram and mass spectra of **6**.

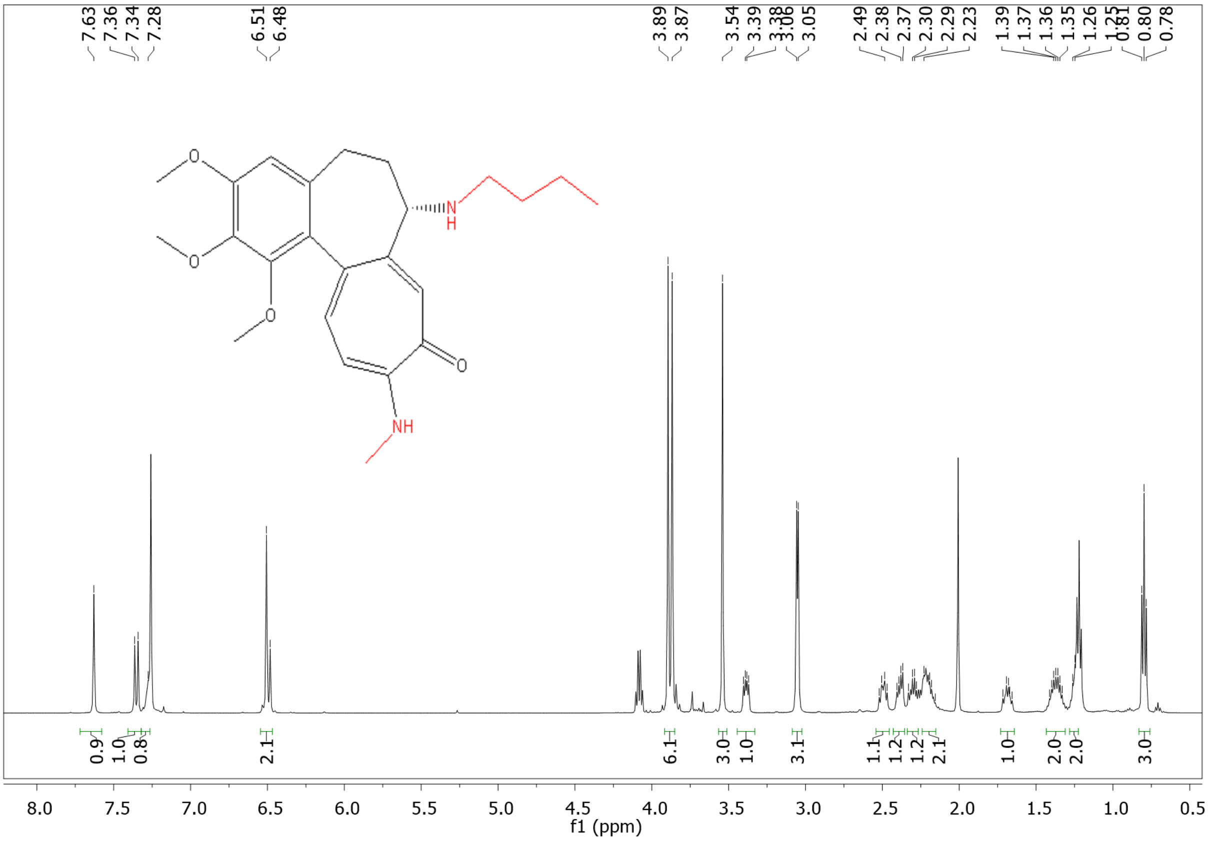

Figure S12. The ^{1}H NMR spectrum of **6** in $CDCl_3$.

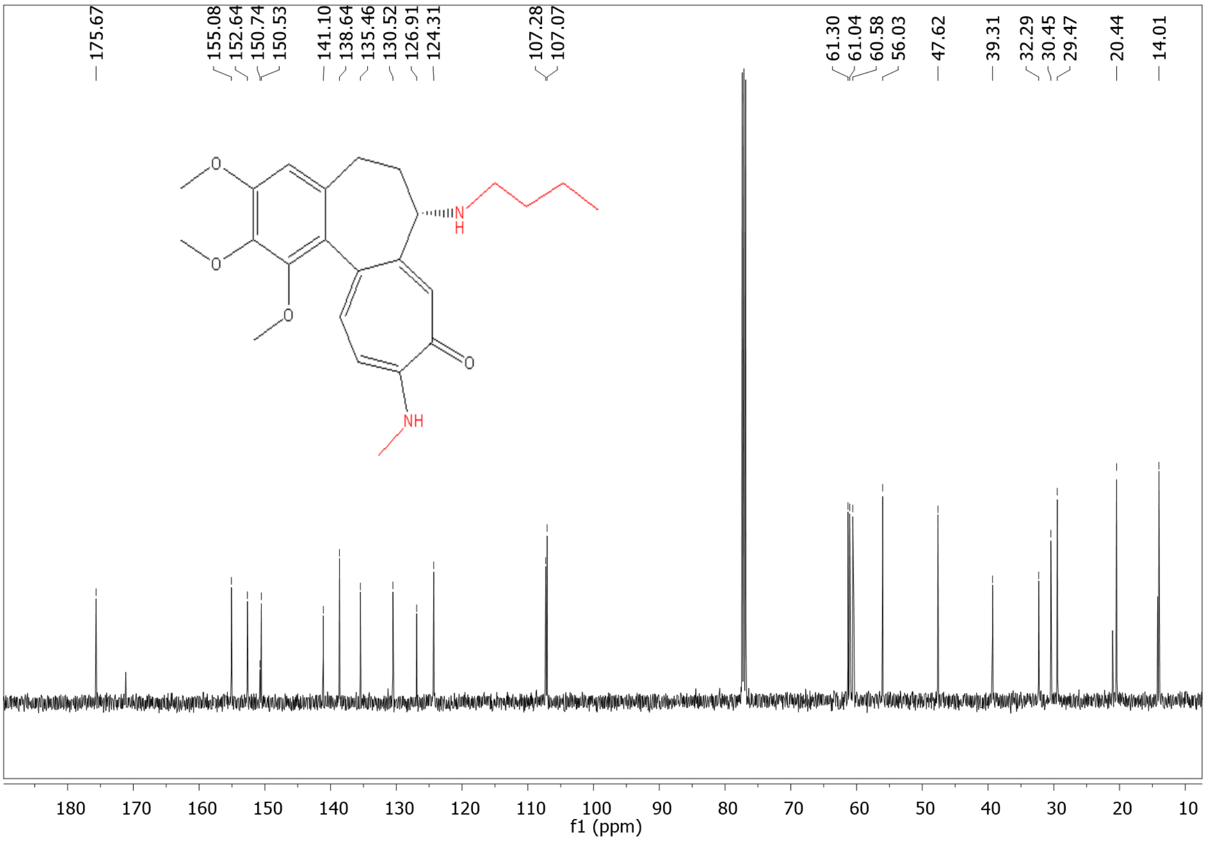

Figure S13. The ^{13}C NMR spectrum of **6** in $CDCl_3$.

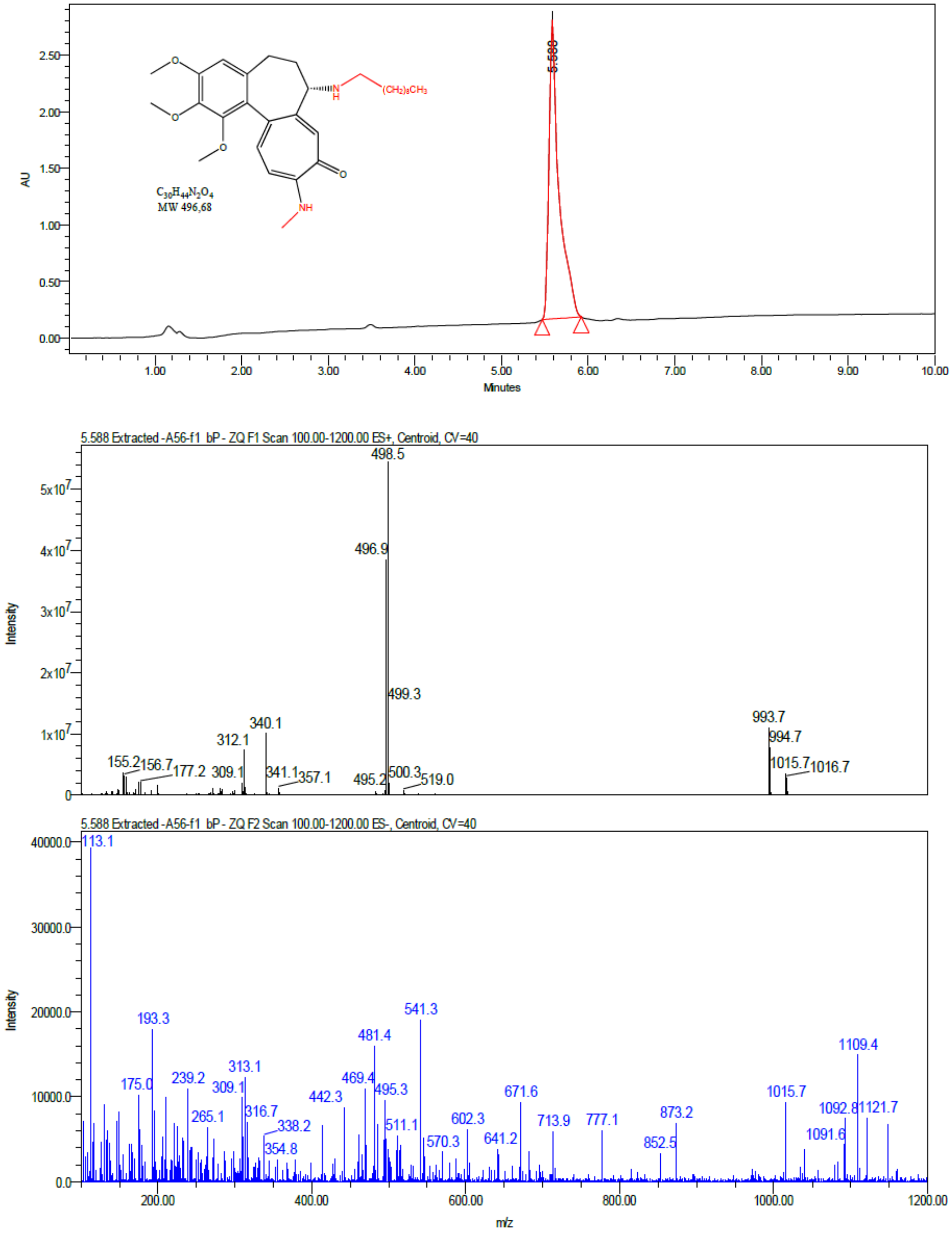

Figure S14. The LC-MS chromatogram and mass spectra of **7**.

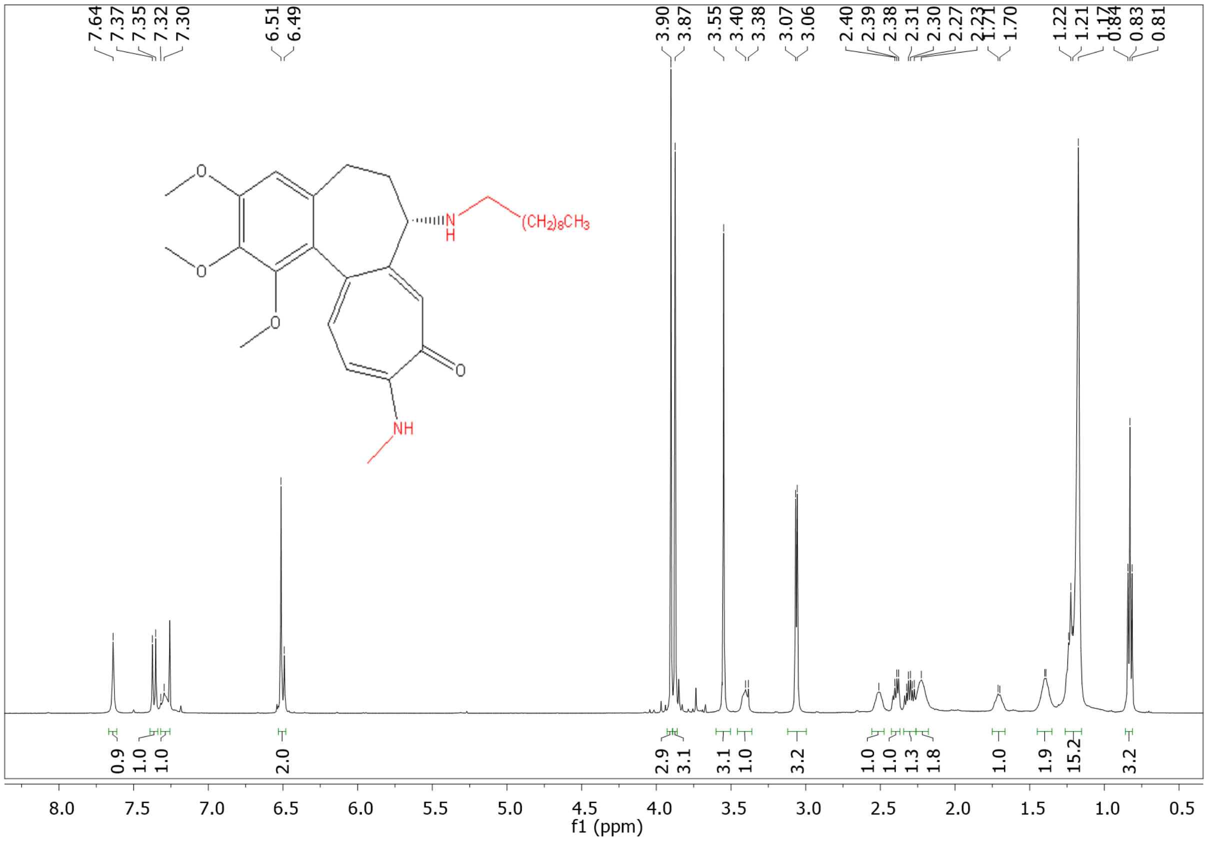

Figure S15. The ^{1}H NMR spectrum of **7** in $CDCl_3$.

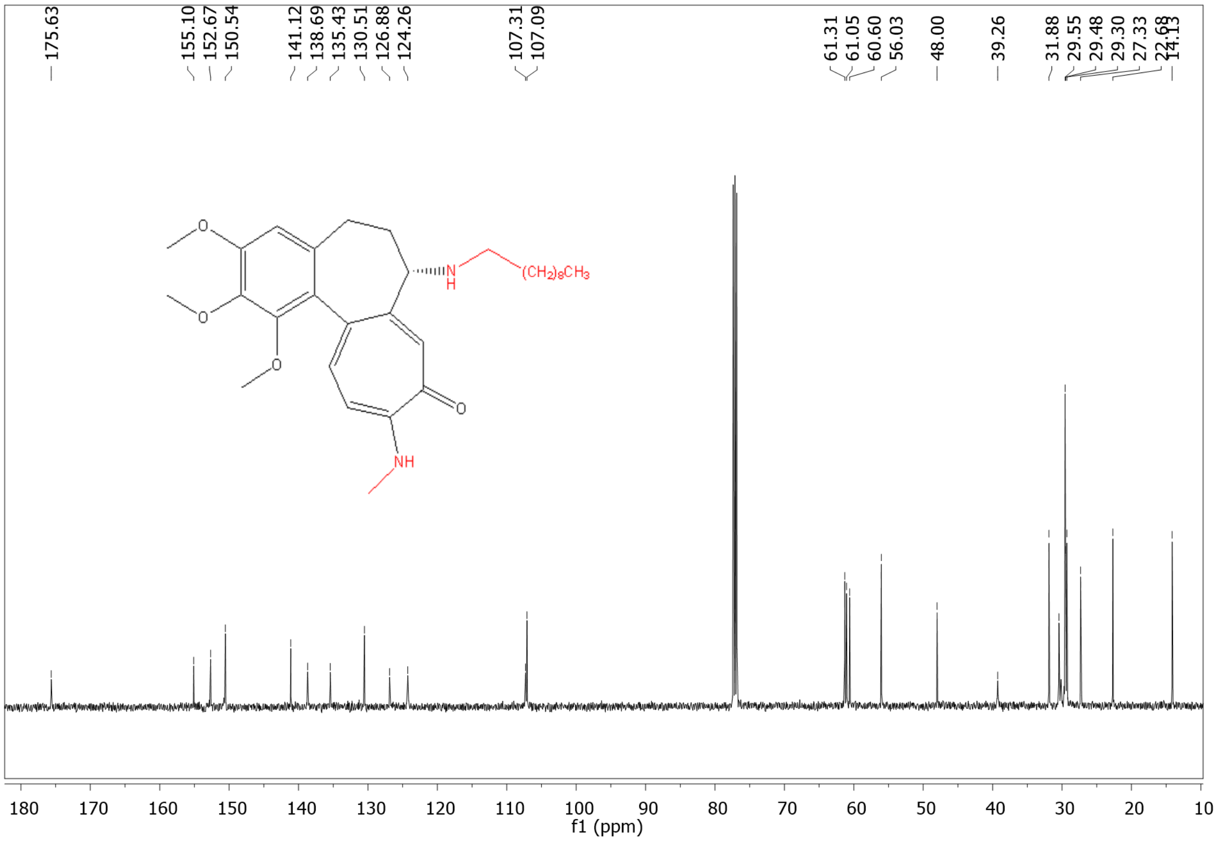

Figure S16. The ^{13}C NMR spectrum of **7** in $CDCl_3$.

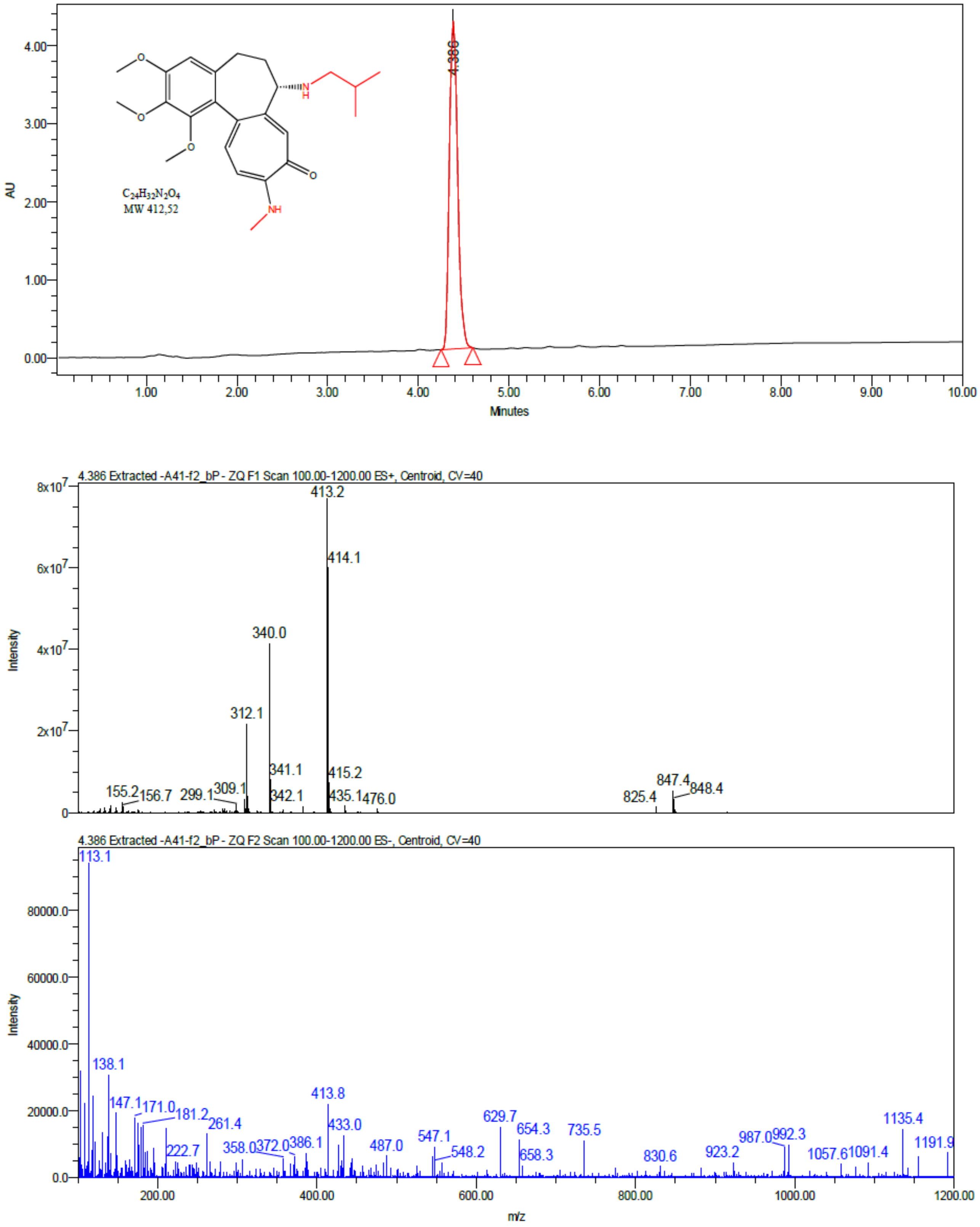

Figure S17. The LC-MS chromatogram and mass spectra of **8**.

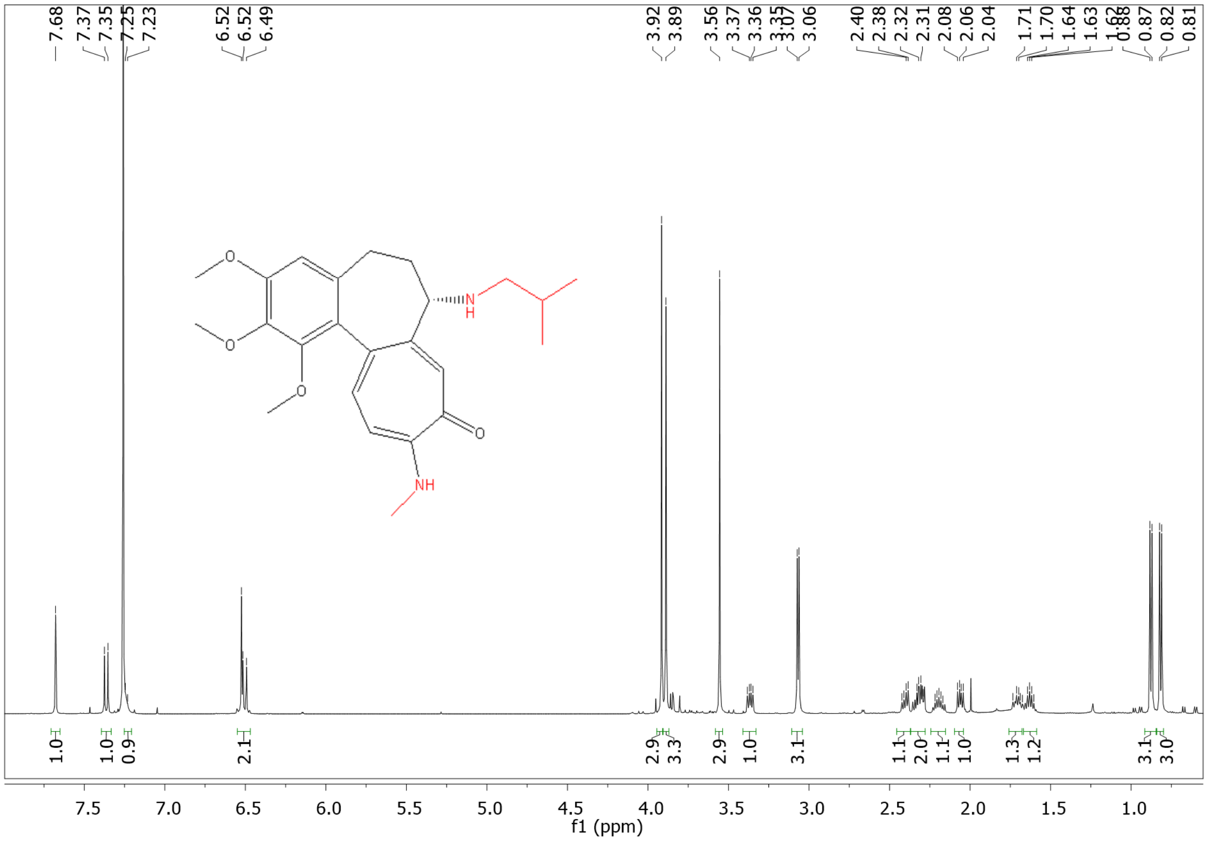

Figure S18. The ^{1}H NMR spectrum of **8** in $CDCl_3$.

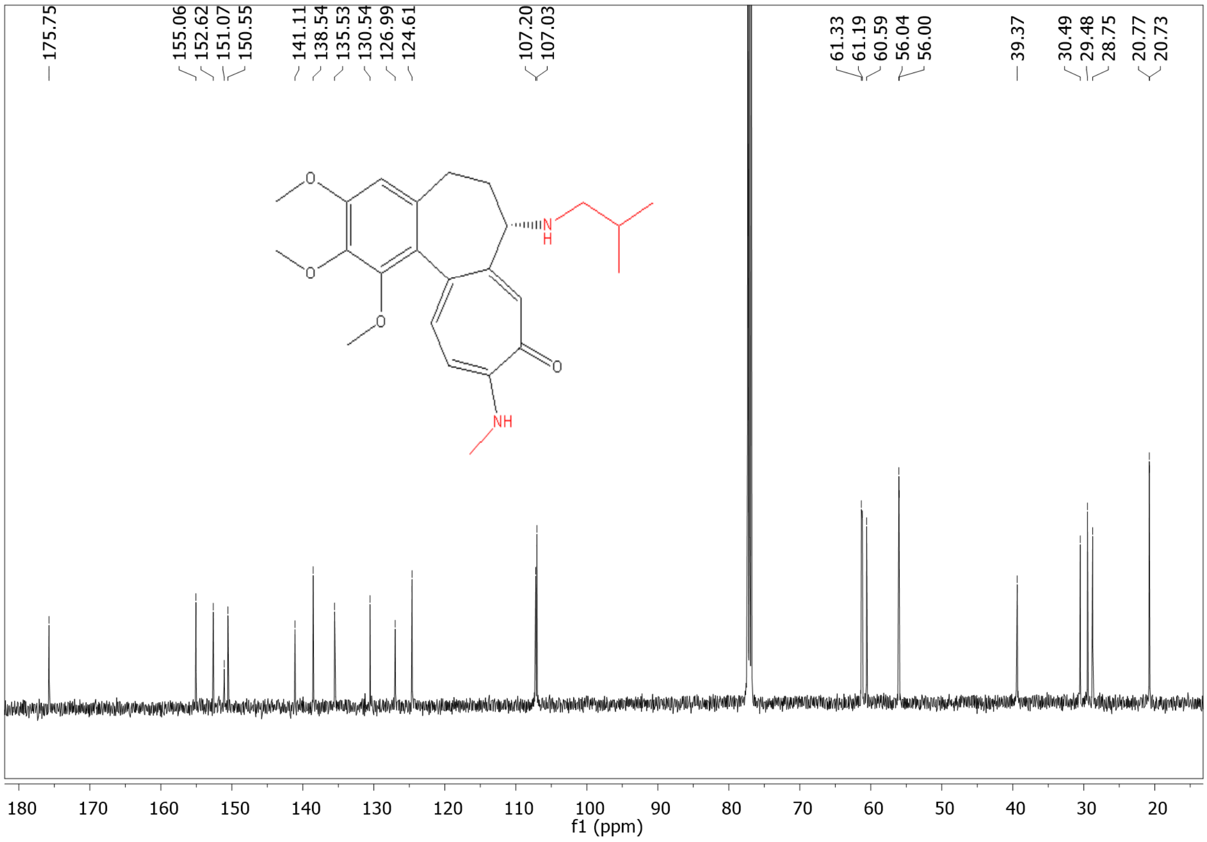

Figure S19. The ^{13}C NMR spectrum of **8** in $CDCl_3$.

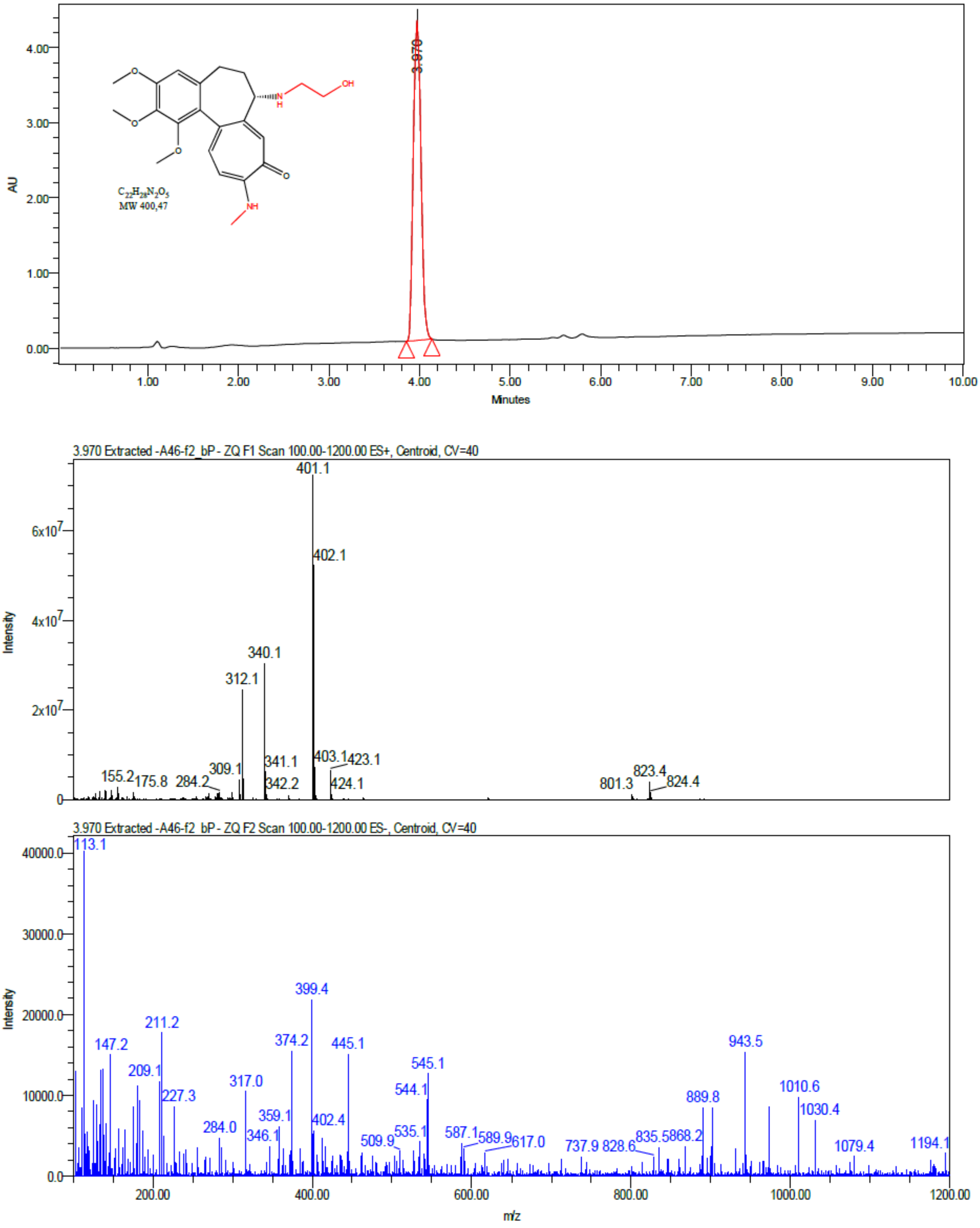

Figure S20. The LC-MS chromatogram and mass spectra of **9**.

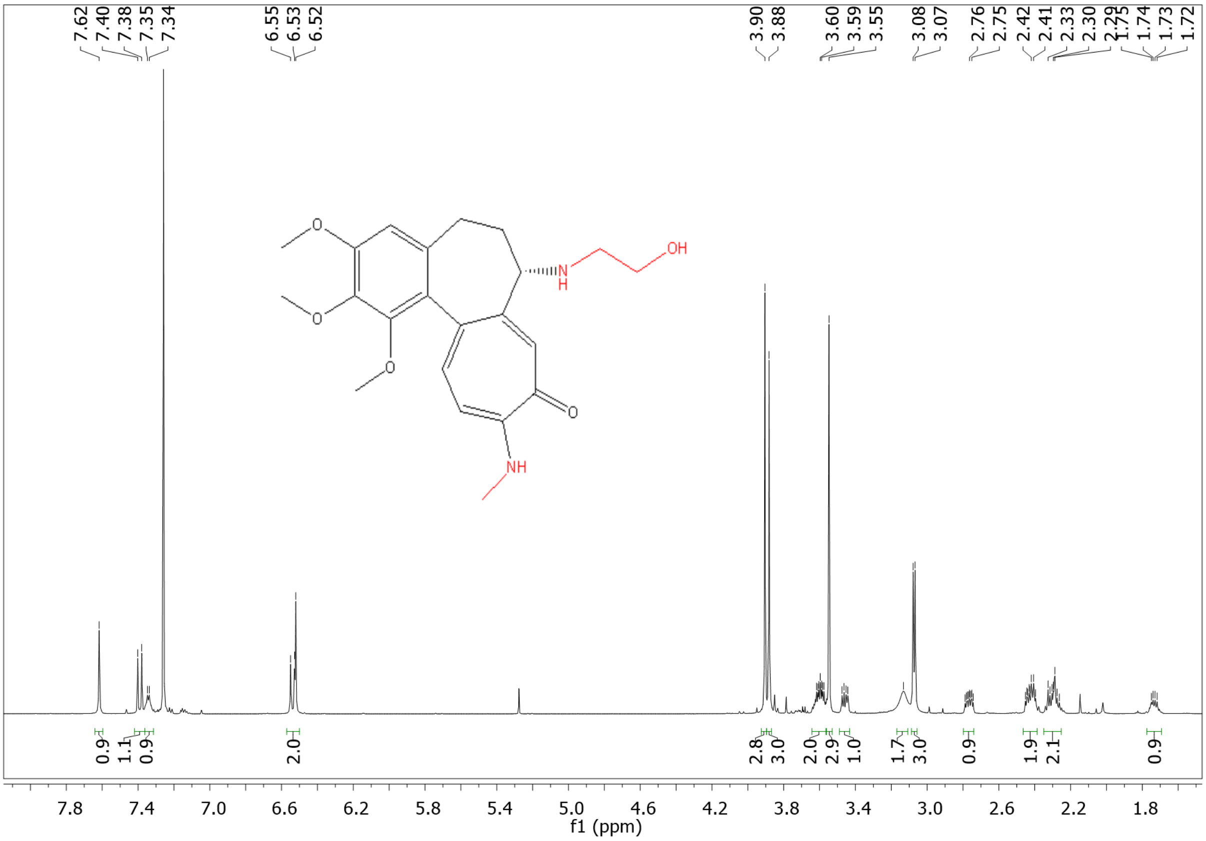

Figure S21. The ^{1}H NMR spectrum of **9** in $CDCl_3$.

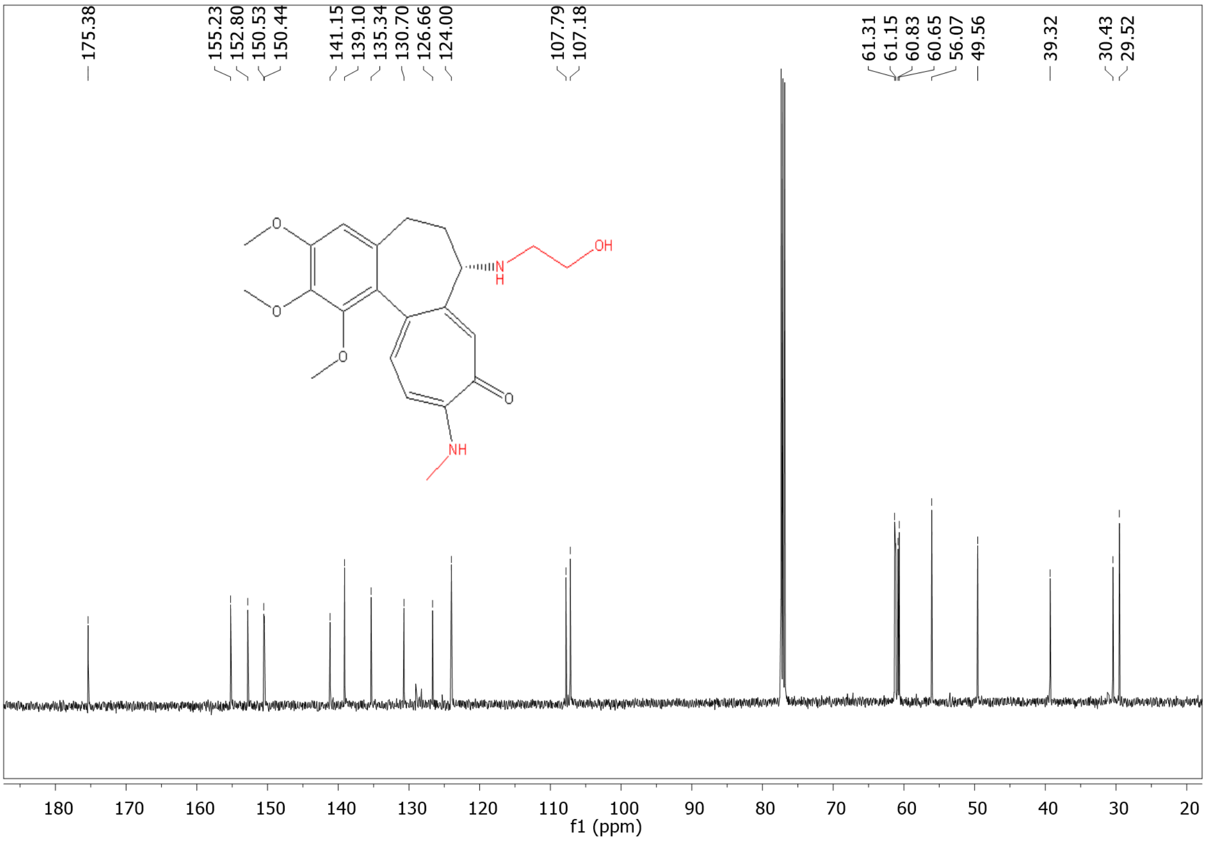

Figure S22. The ^{13}C NMR spectrum of **9** in $CDCl_3$.

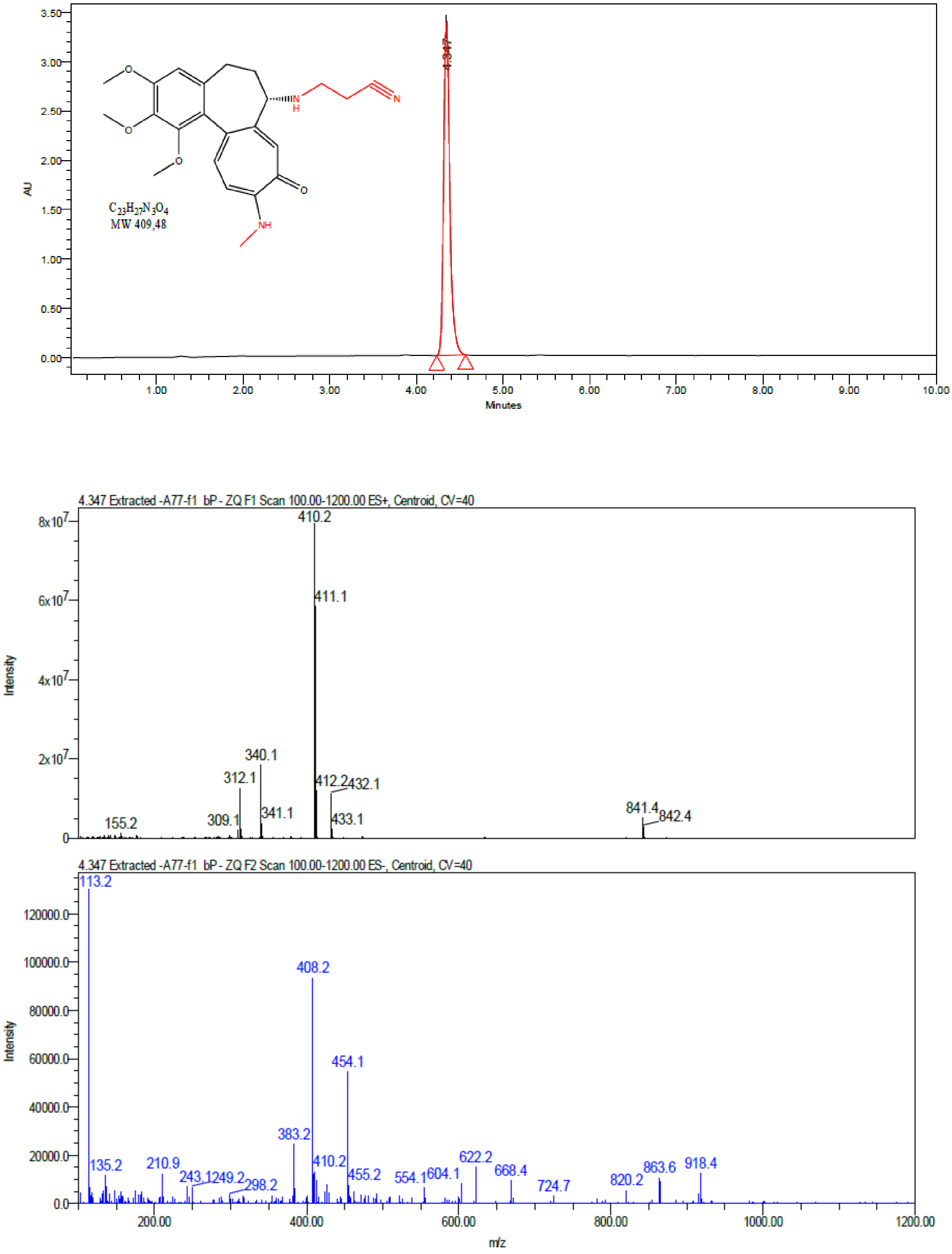

Figure S23. The LC-MS chromatogram and mass spectra of **10**.

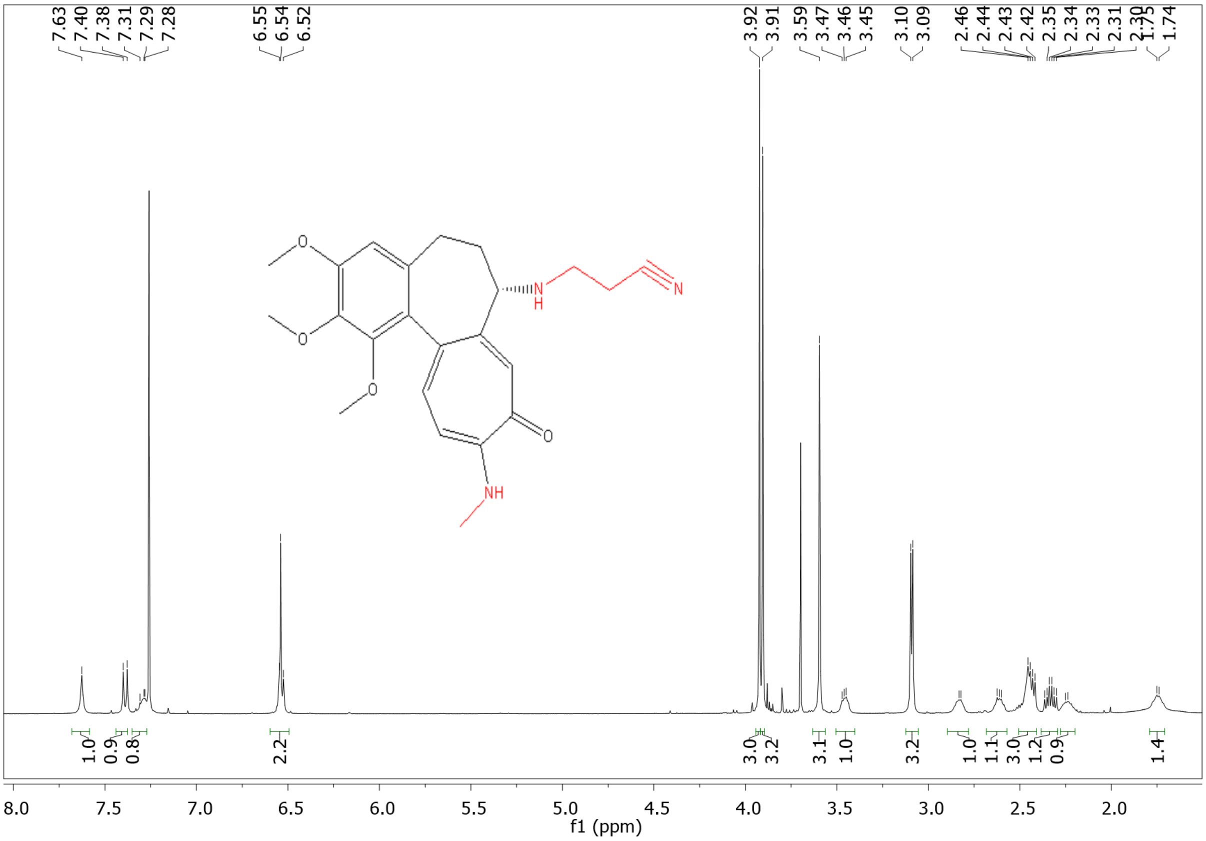

Figure S24. The ^{1}H NMR spectrum of **10** in $CDCl_3$.

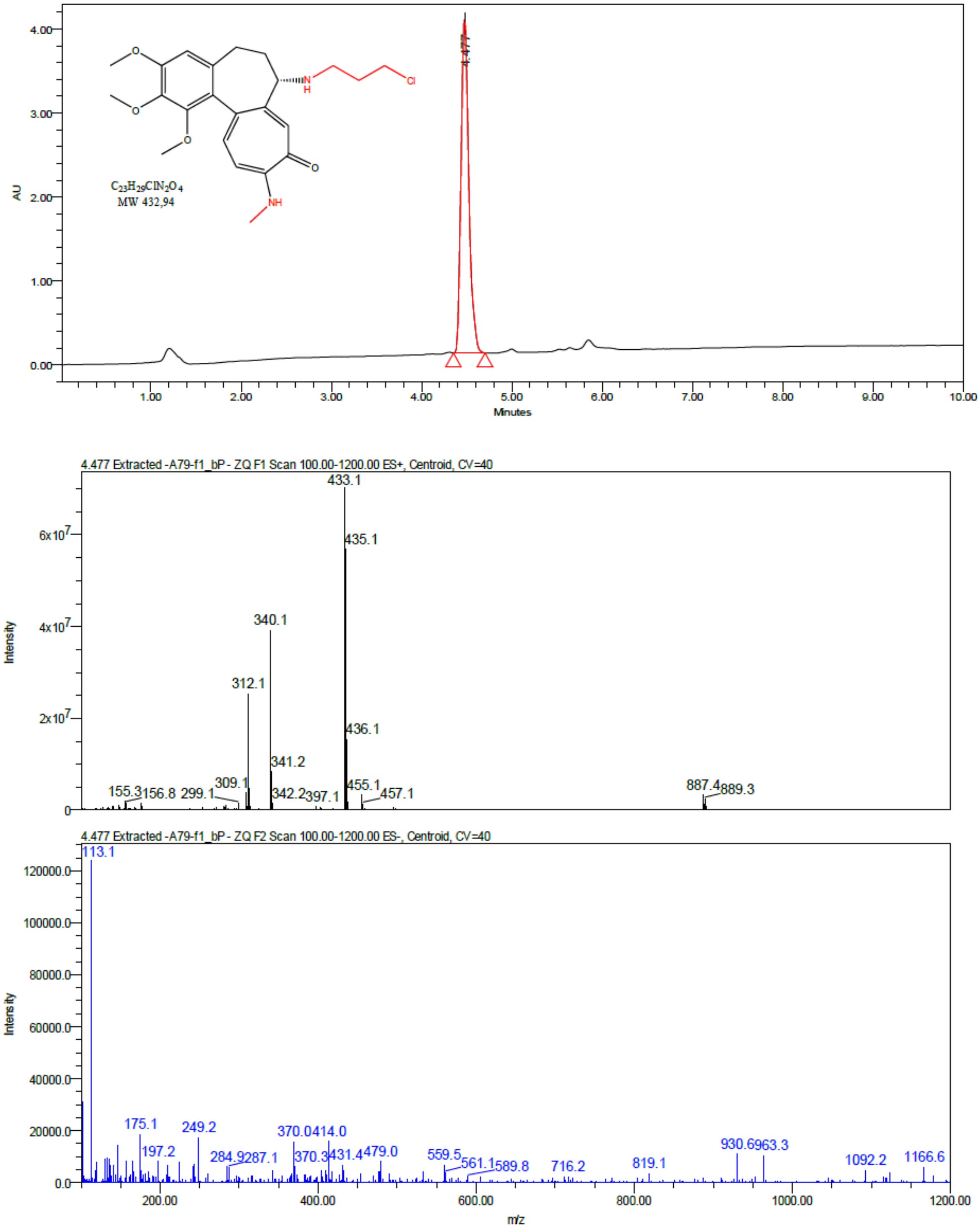

Figure S25. The LC-MS chromatogram and mass spectra of **11**.

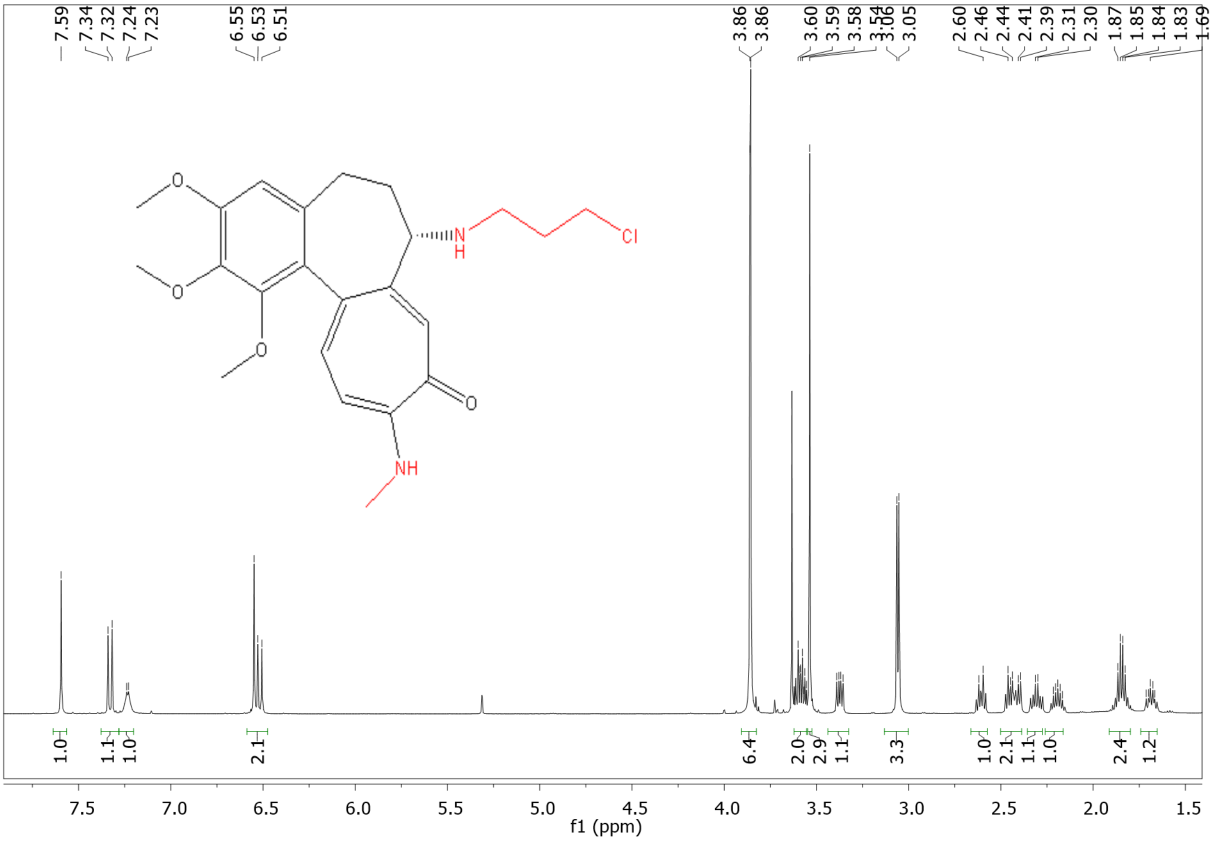

Figure S26. The ^{1}H NMR spectrum of **11** in CD_2Cl_2.

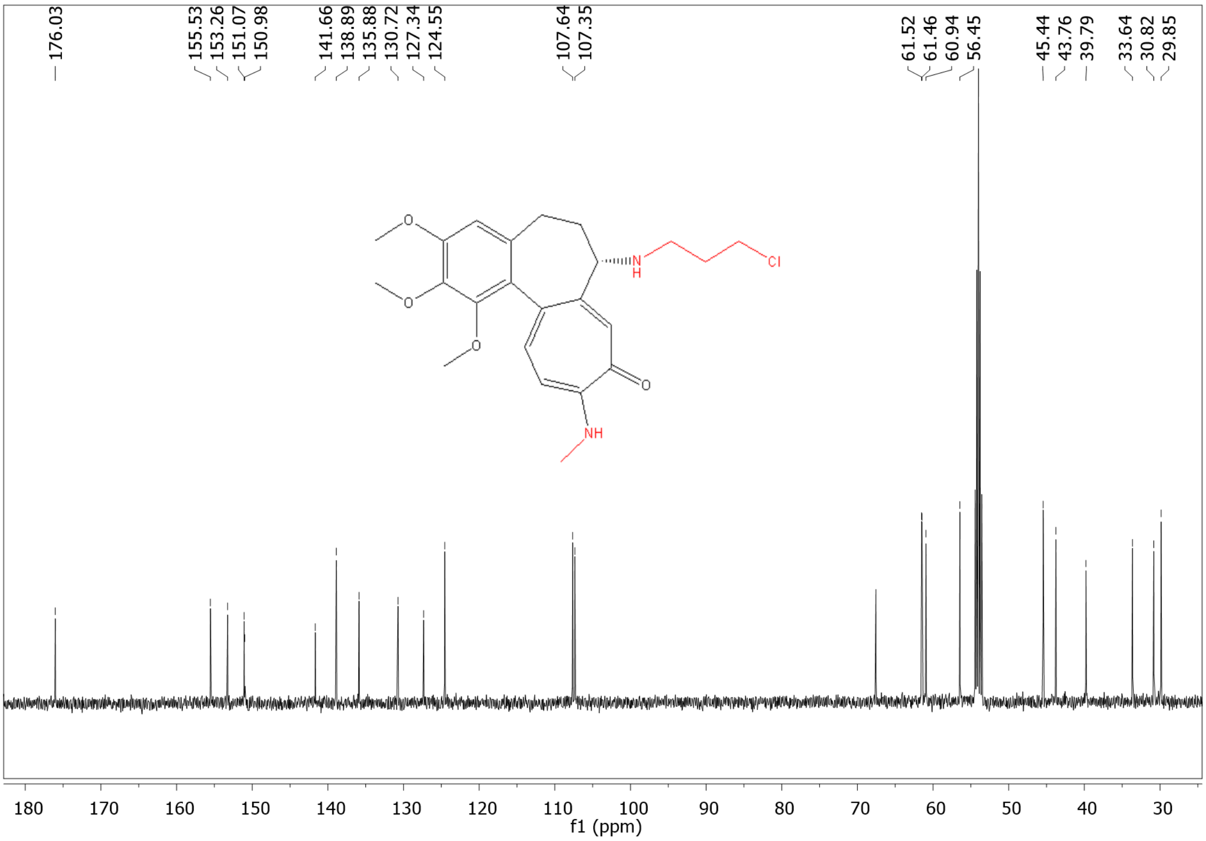

Figure S27. The ^{13}C NMR spectrum of **11** in CD_2Cl_2.

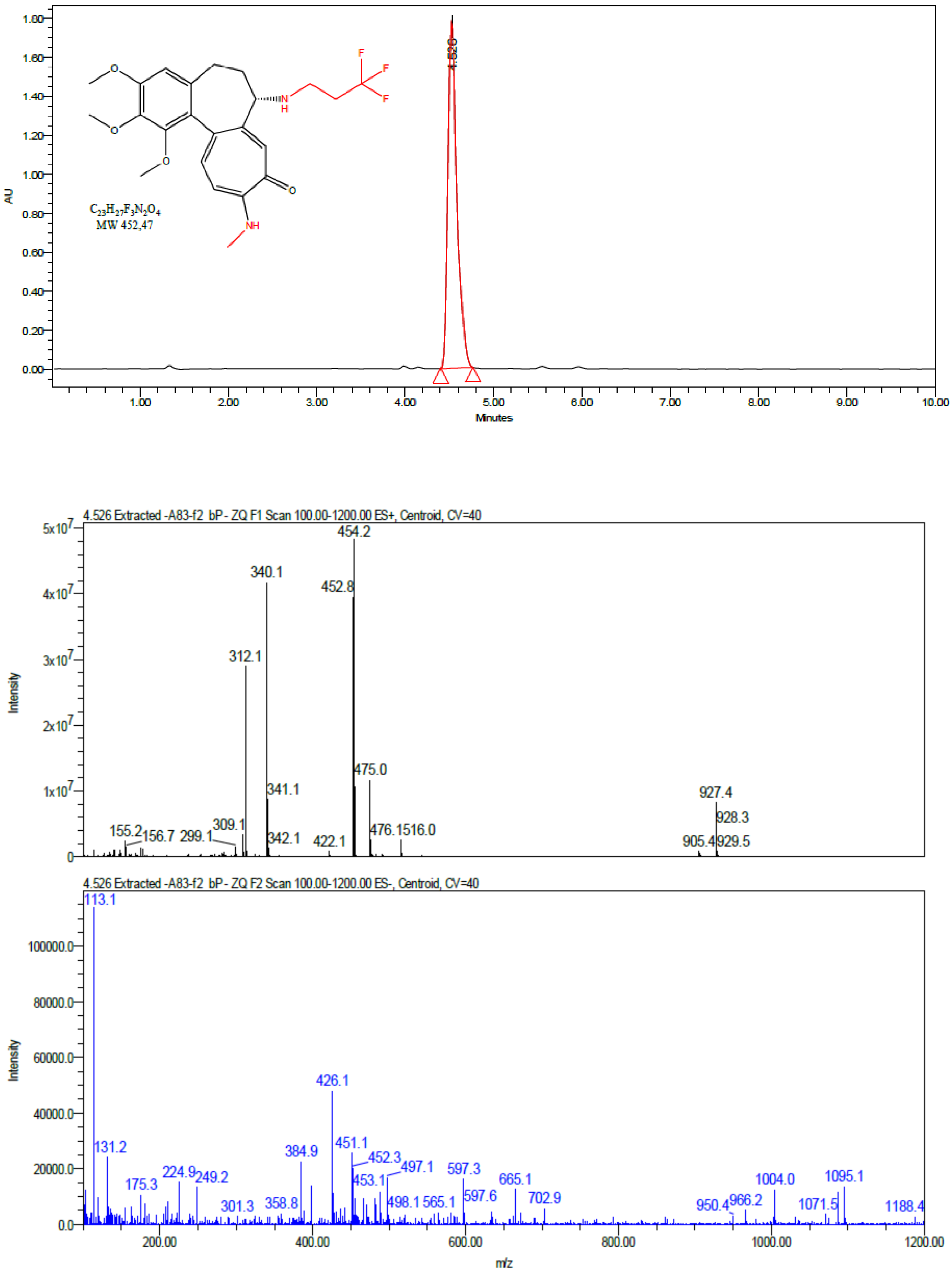

Figure S28. The LC-MS chromatogram and mass spectra of **12**.

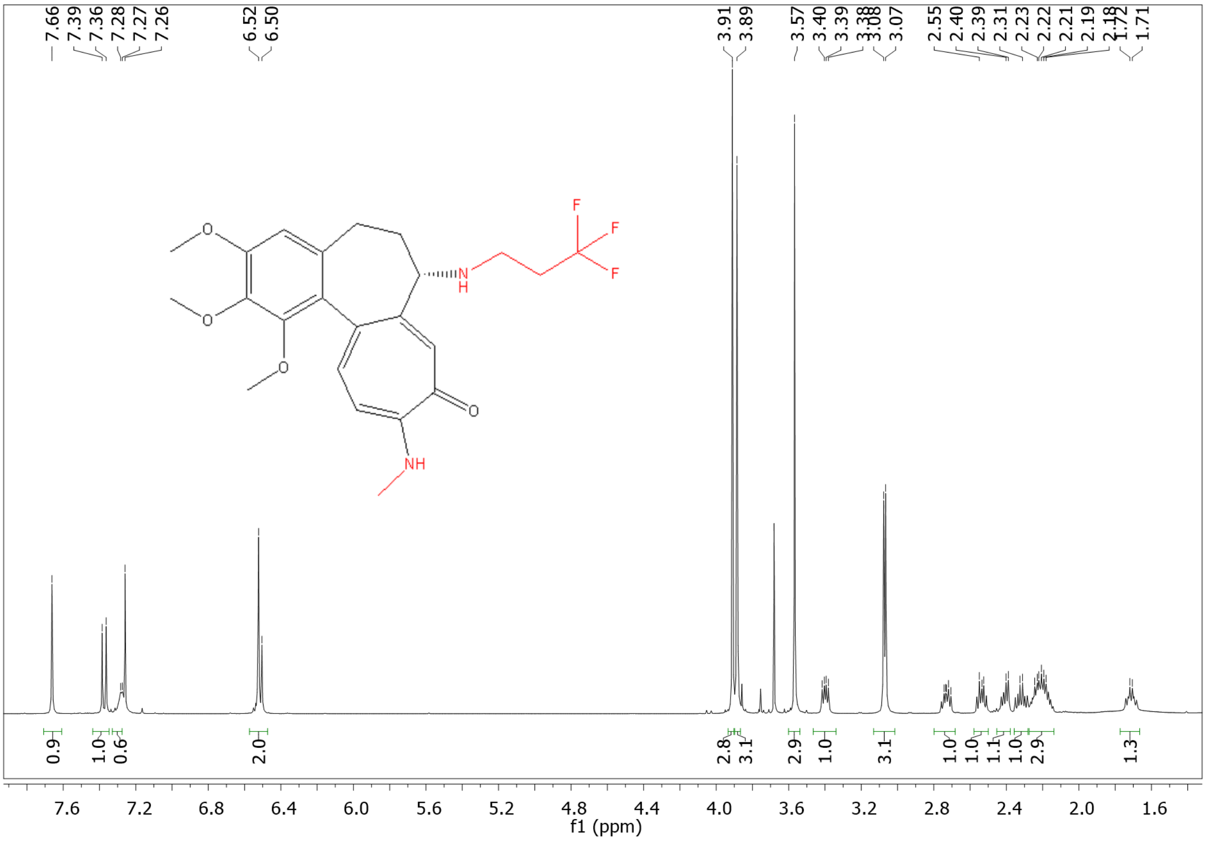

Figure S29. The ^{1}H NMR spectrum of **12** in $CDCl_3$.

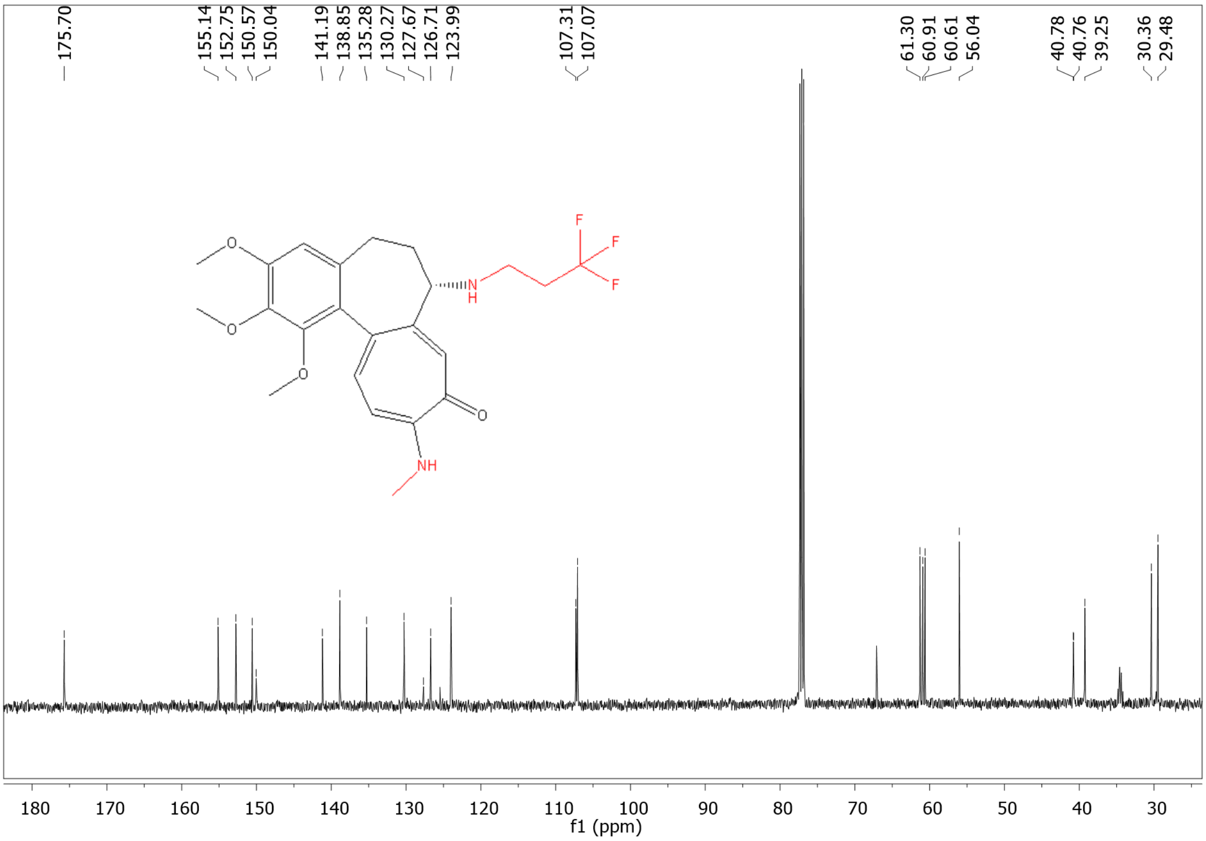

Figure S30. The ^{13}C NMR spectrum of **12** in $CDCl_3$.

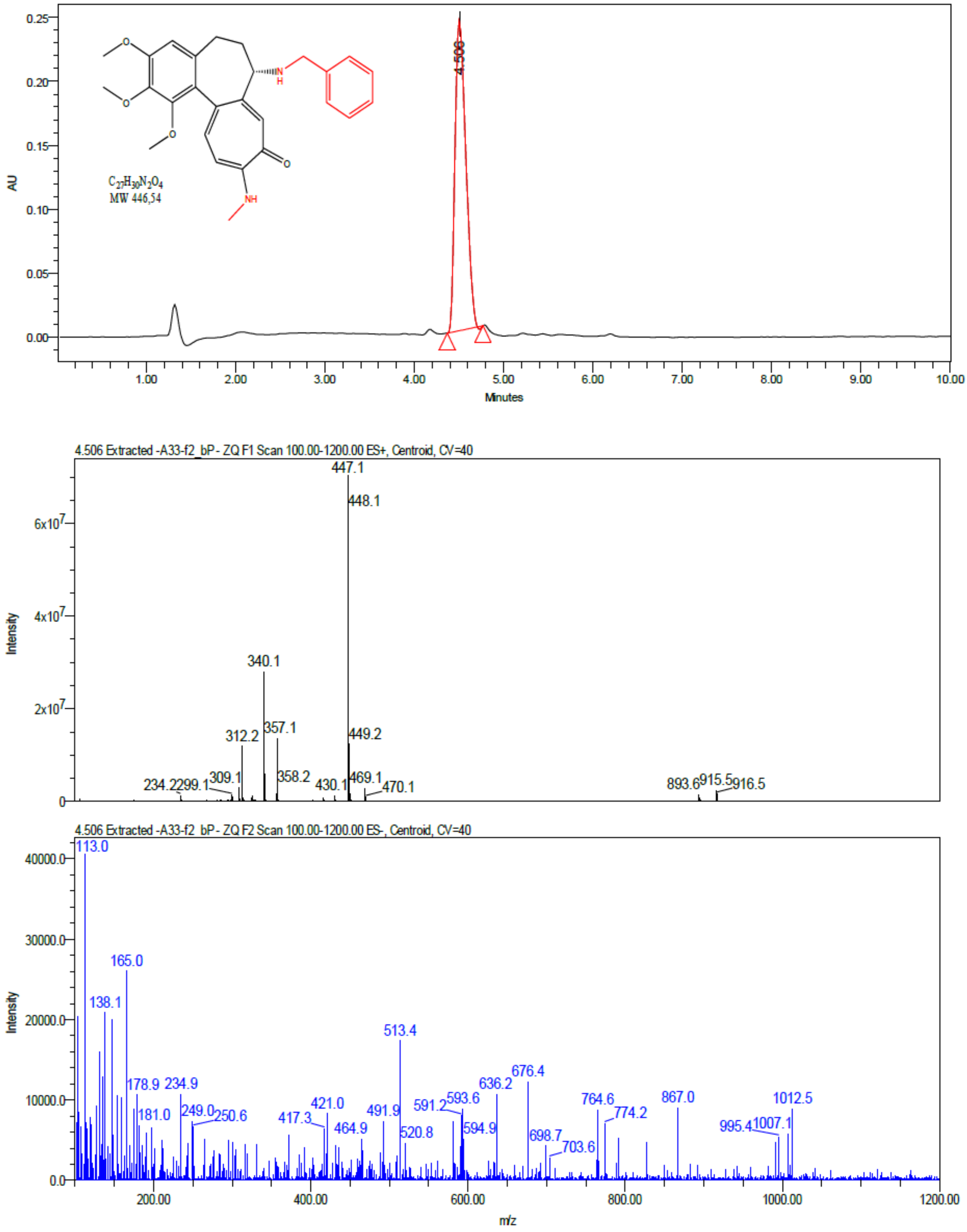

Figure S31. The LC-MS chromatogram and mass spectra of **13**.

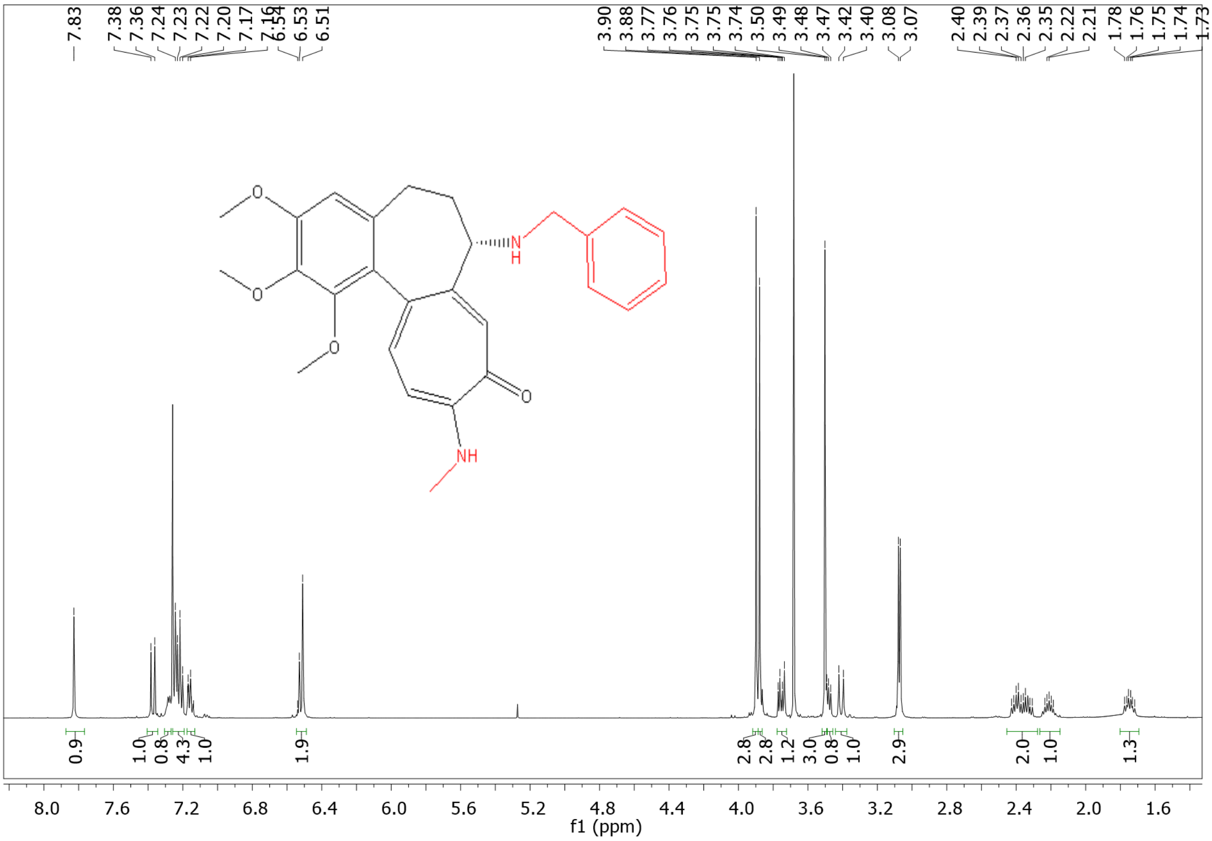

Figure S32. The ^{1}H NMR spectrum of **13** in $CDCl_3$.

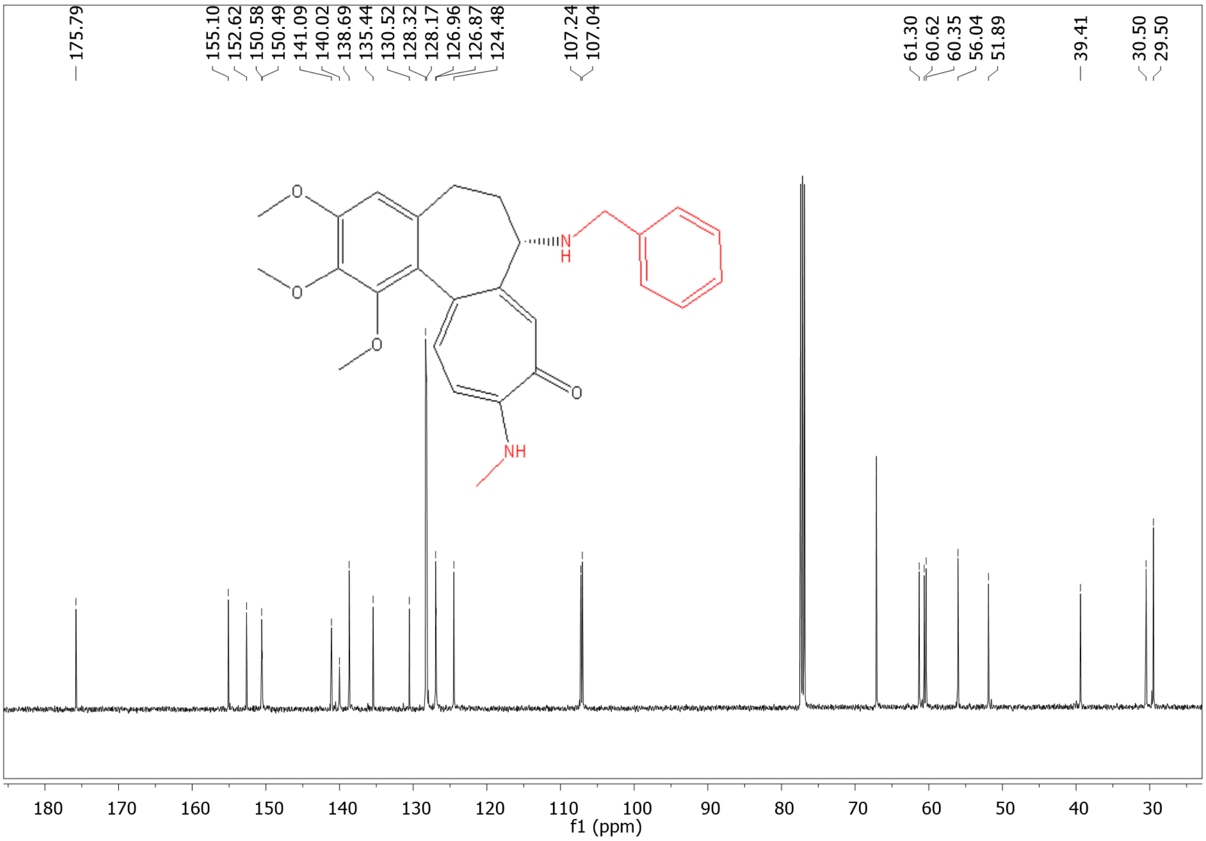

Figure S33. The ^{13}C NMR spectrum of **13** in $CDCl_3$.

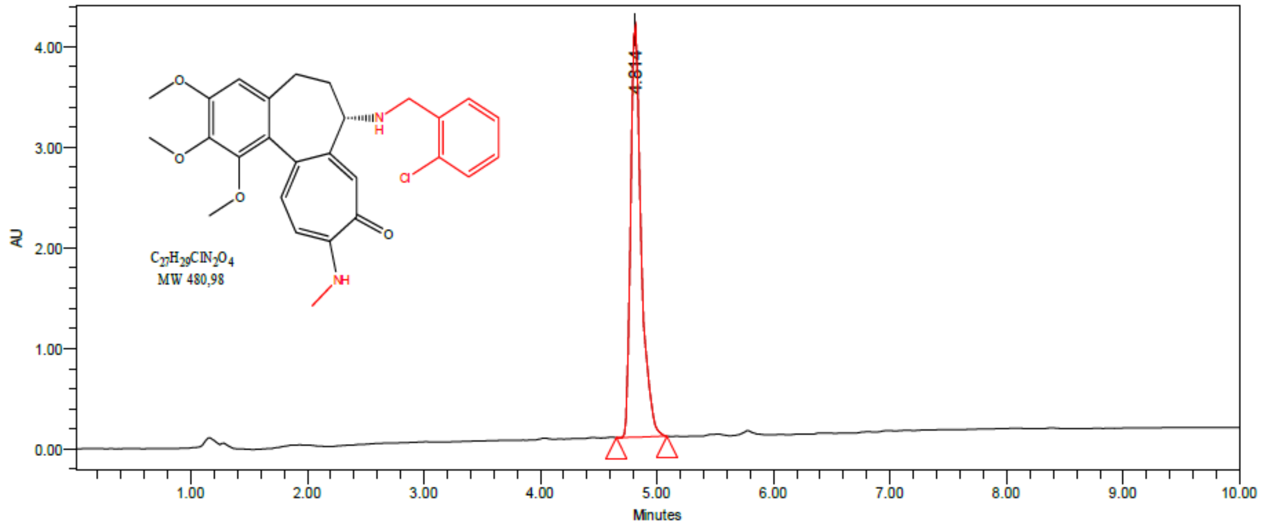

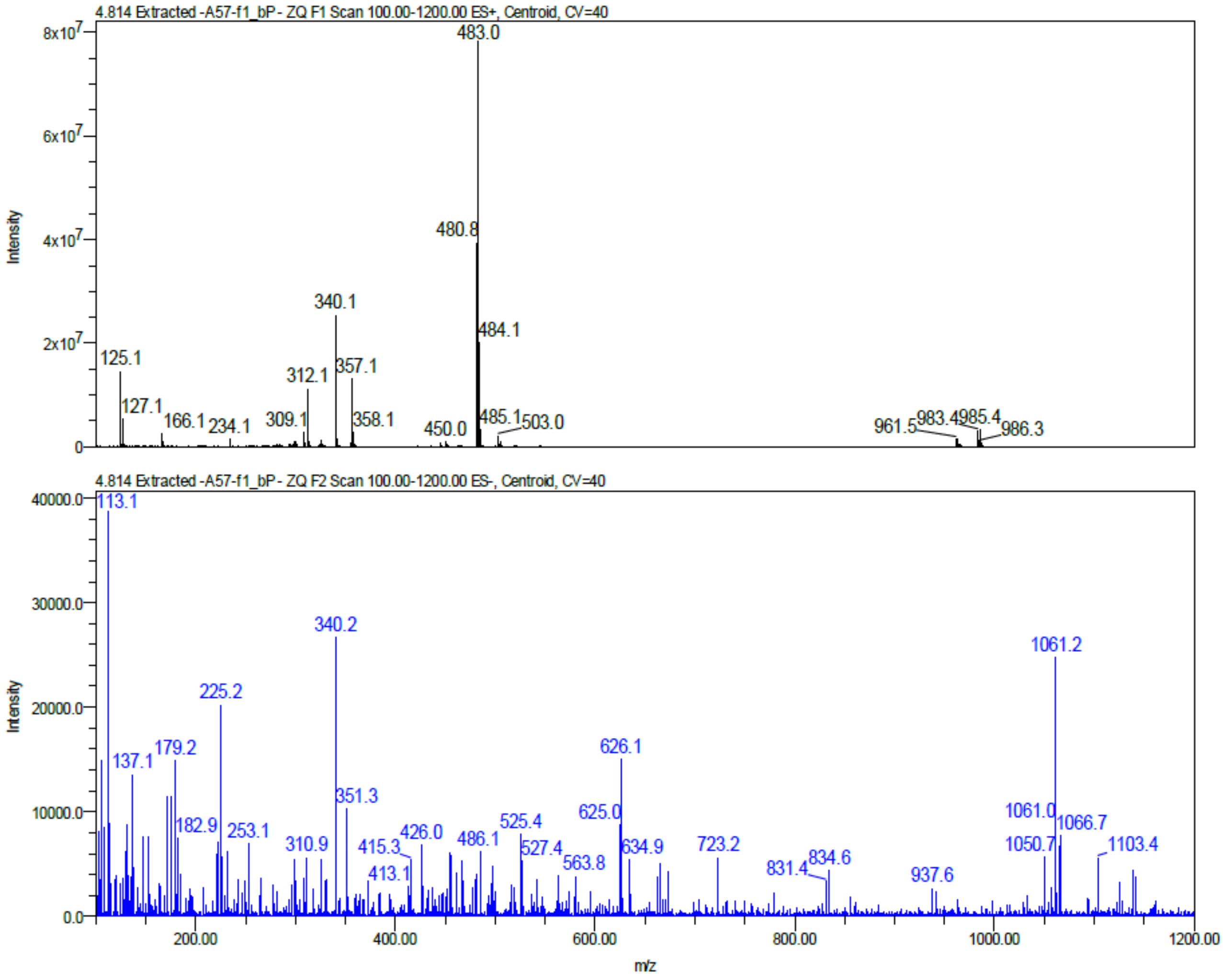

Figure S34. The LC-MS chromatogram and mass spectra of **14**.

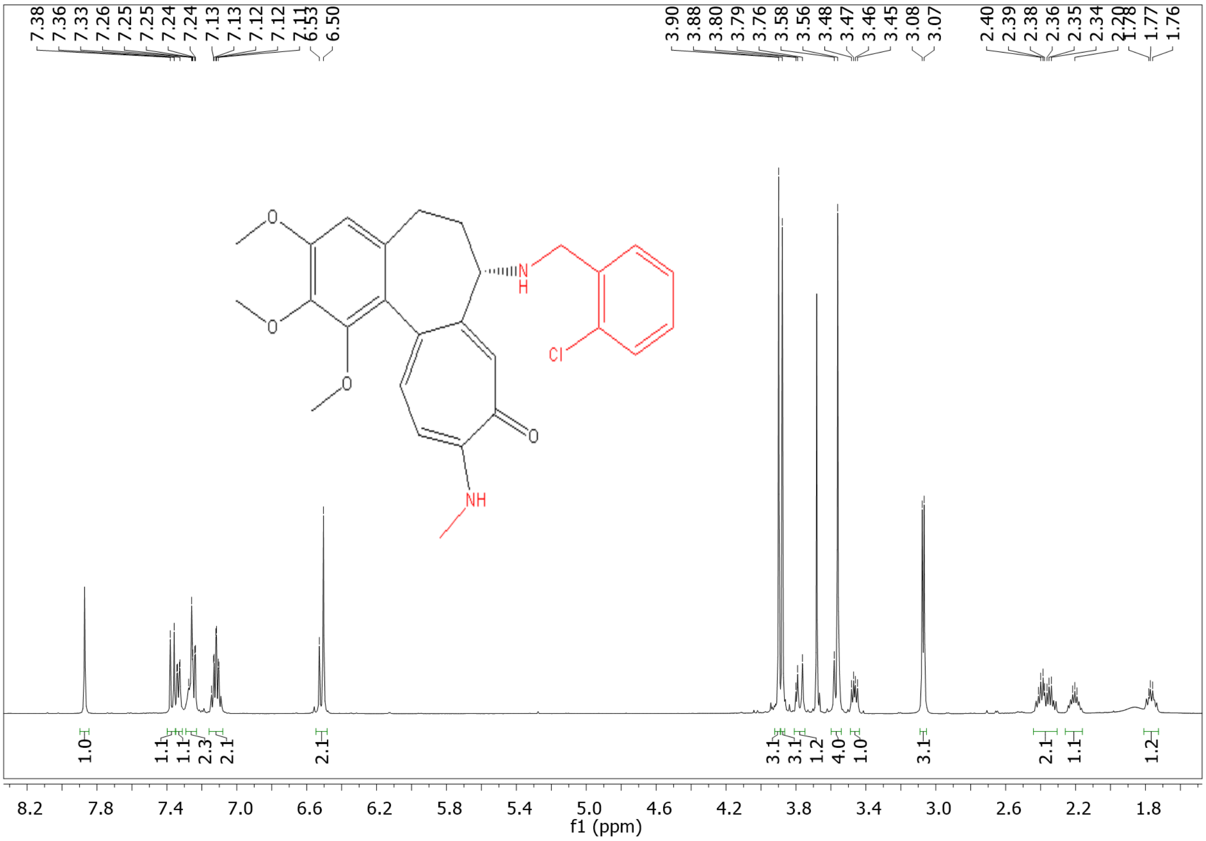

Figure S35. The ^{1}H NMR spectrum of **14** in $CDCl_3$.

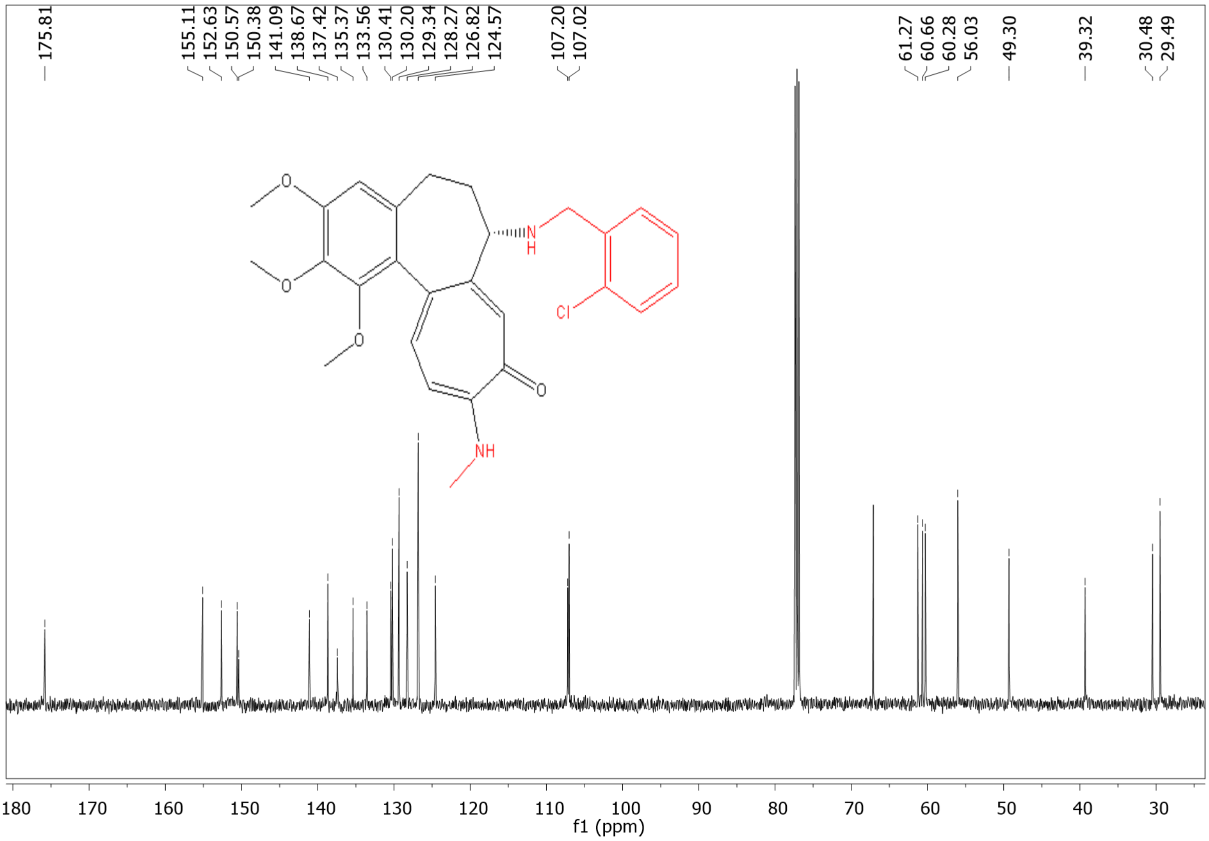

Figure S36. The ^{13}C NMR spectrum of **14** in $CDCl_3$.

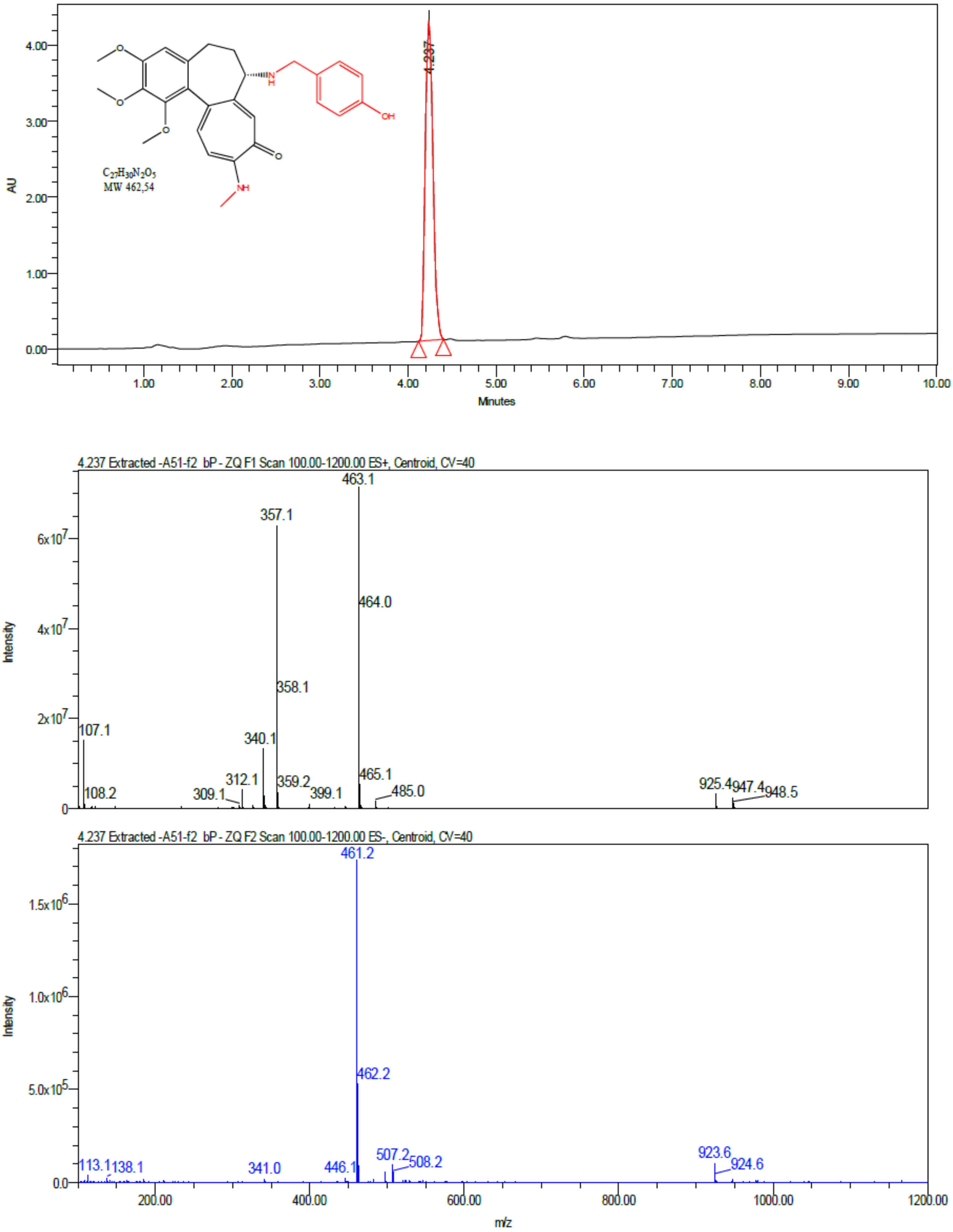

Figure S37. The LC-MS chromatogram and mass spectra of **15**.

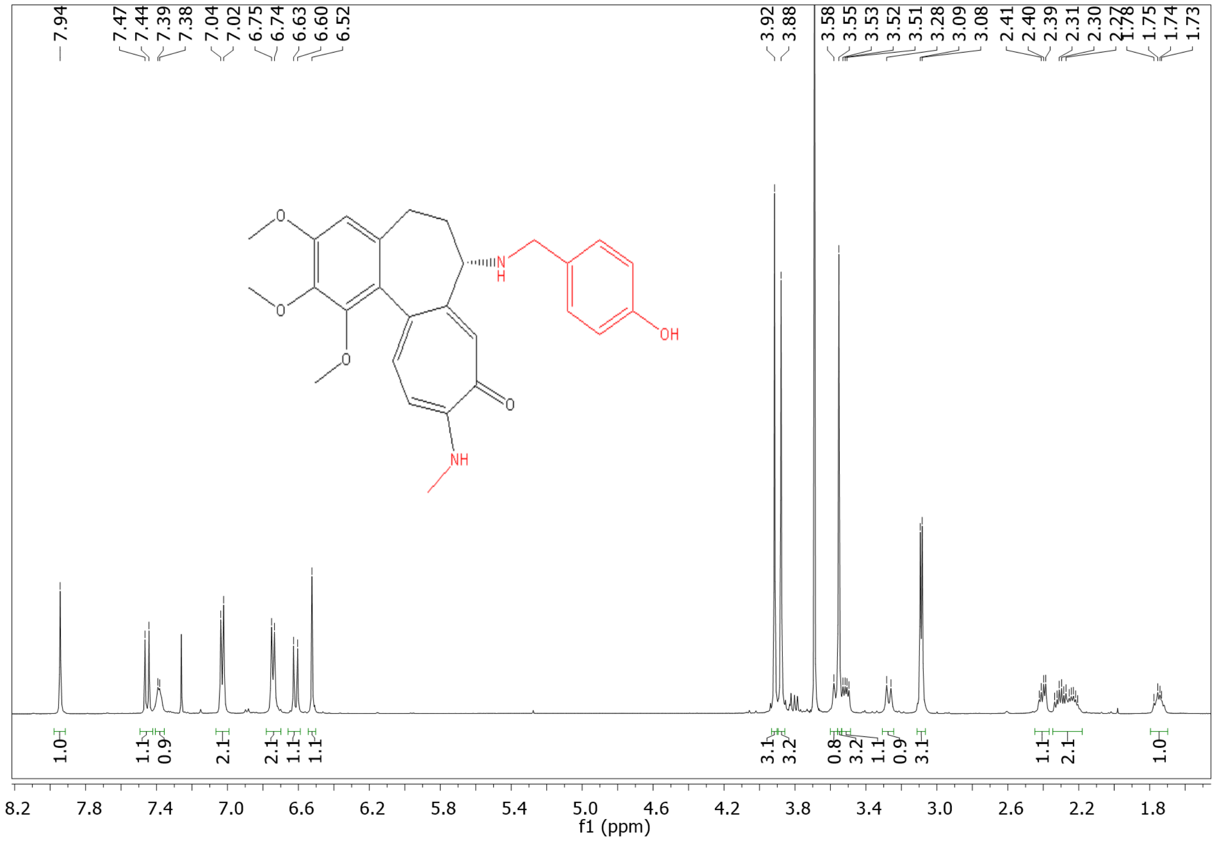

Figure S38. The ^{1}H NMR spectrum of **15** in $CDCl_3$.

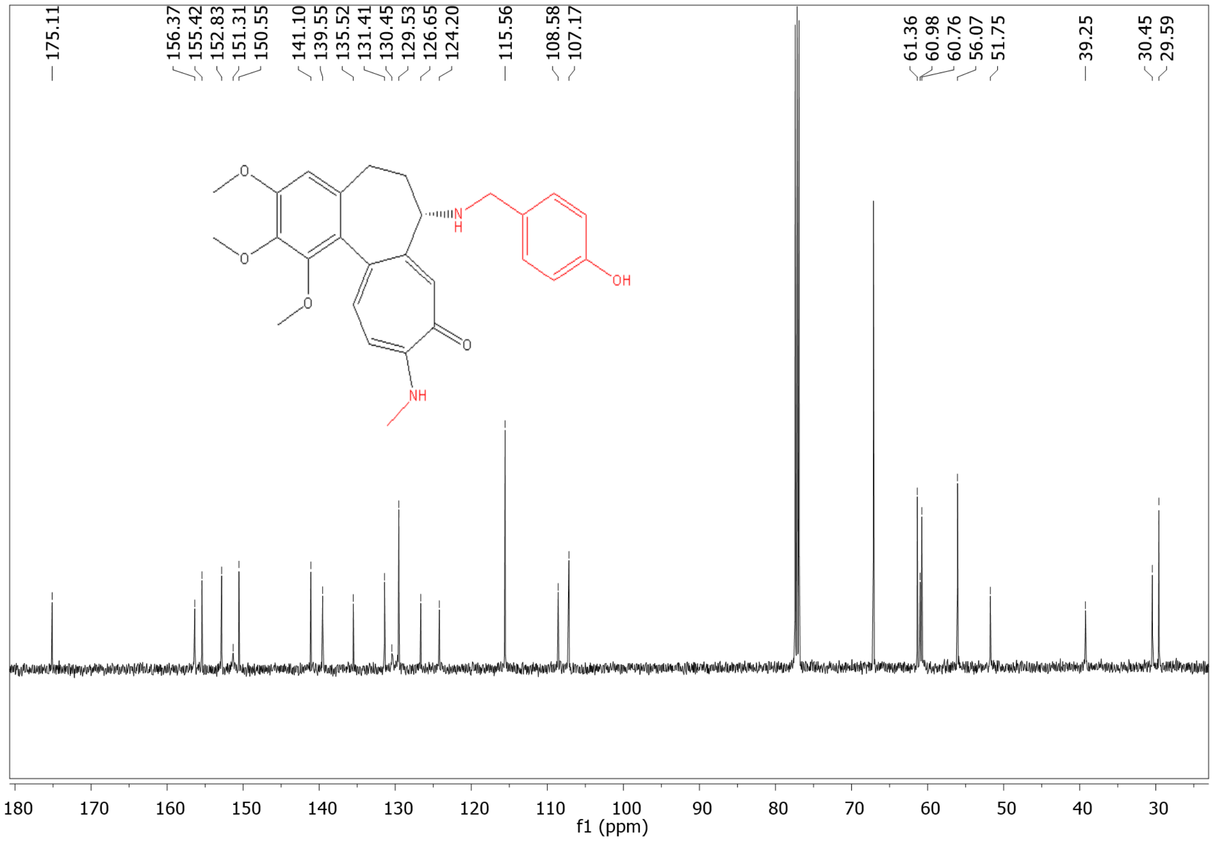

Figure S39. The ^{13}C NMR spectrum of **15** in $CDCl_3$.

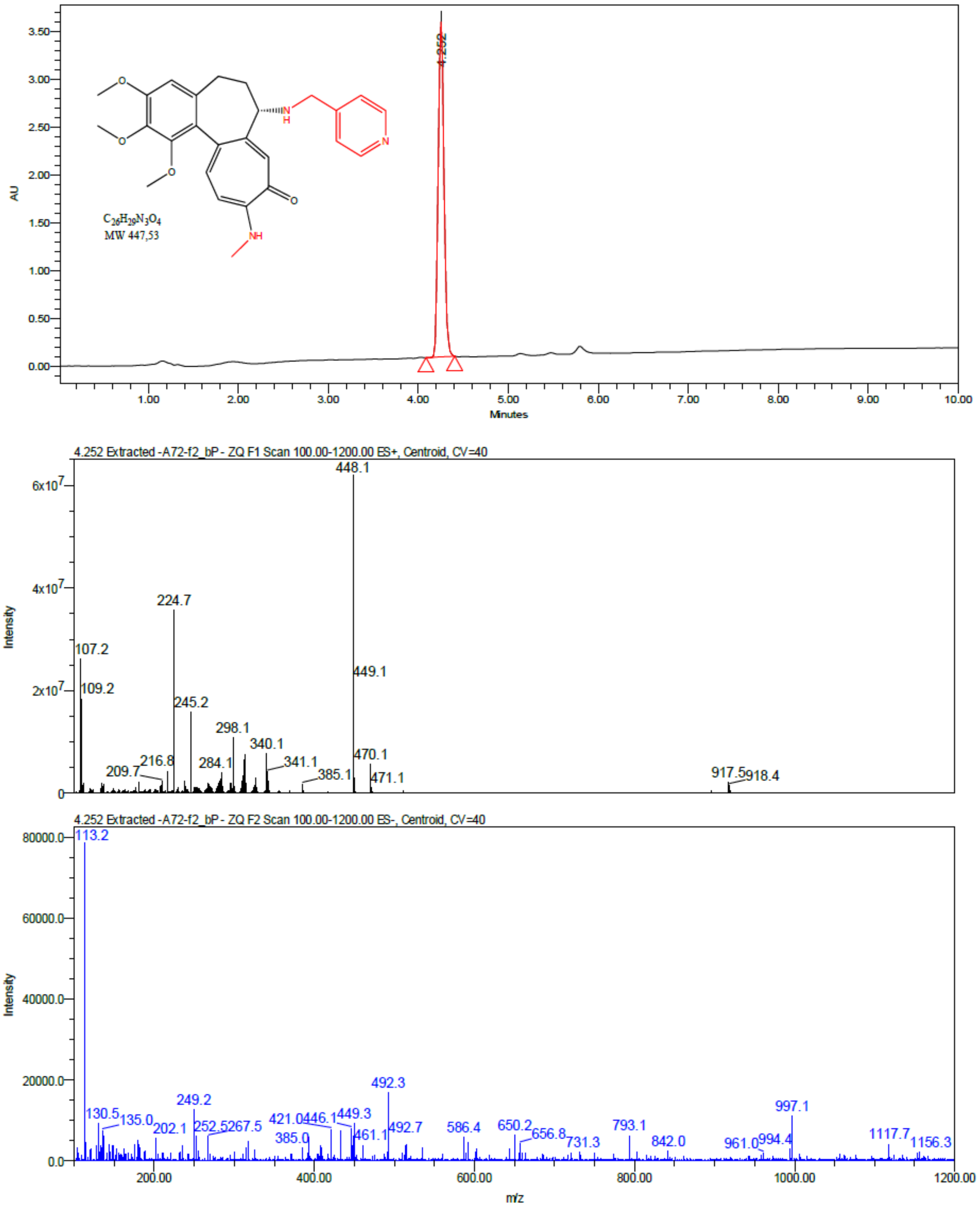

Figure S40. The LC-MS chromatogram and mass spectra of **16**.

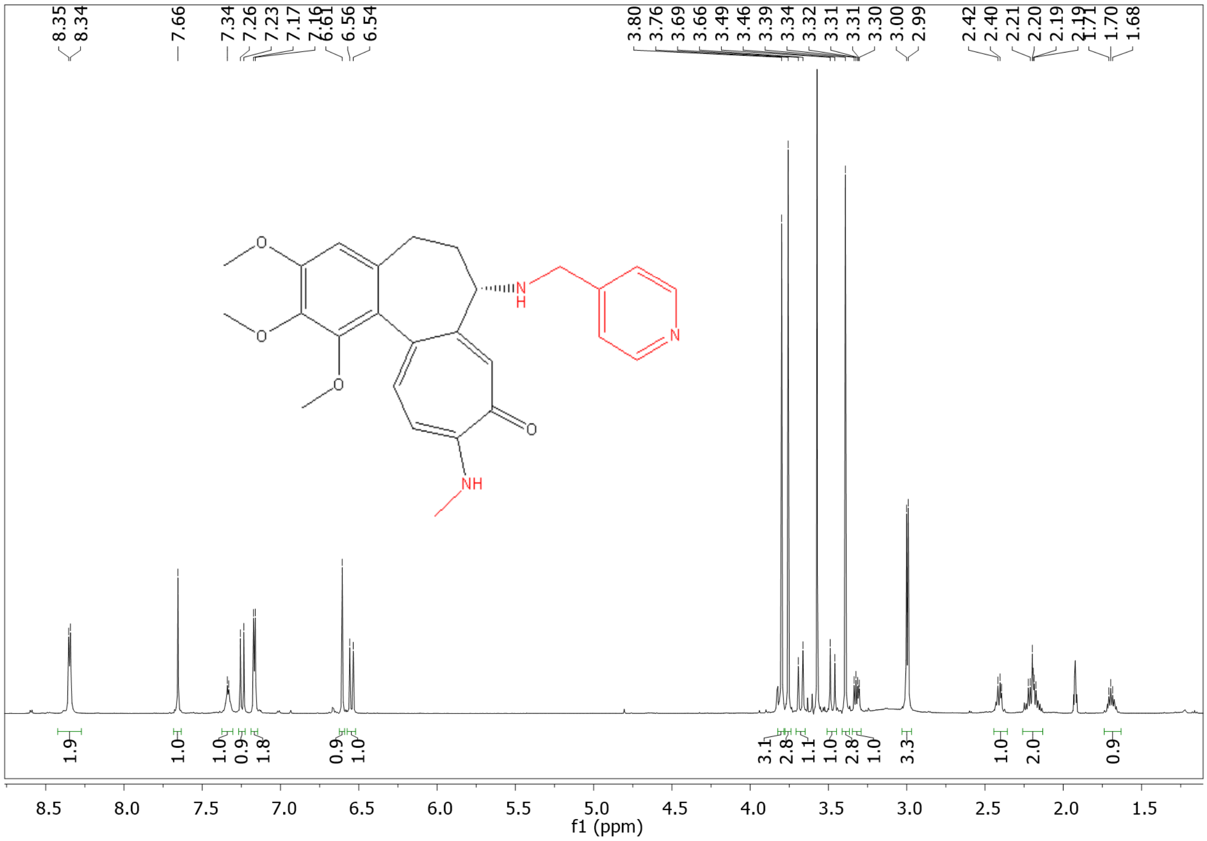

Figure S41. The ^{1}H NMR spectrum of **16** in CD_3CN.

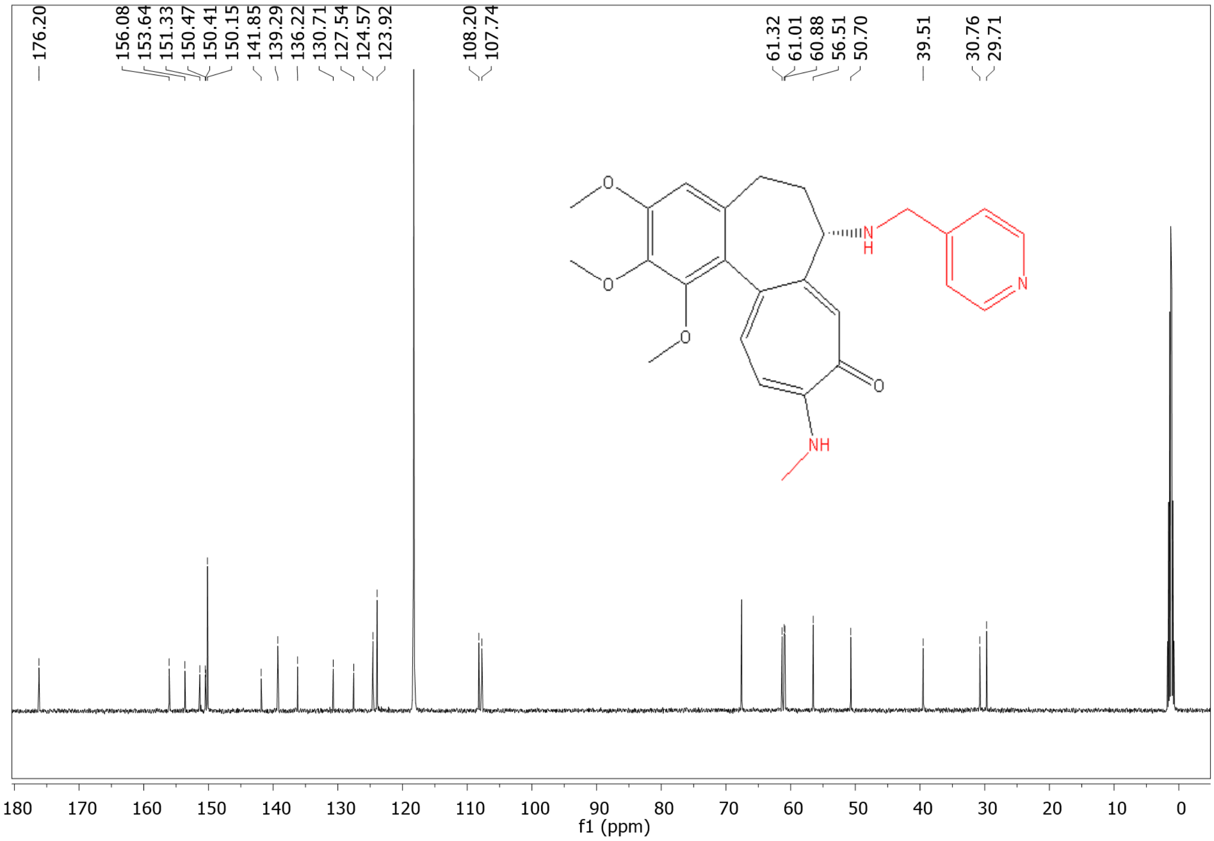

Figure S42. The ^{13}C NMR spectrum of **16** in CD_3CN.

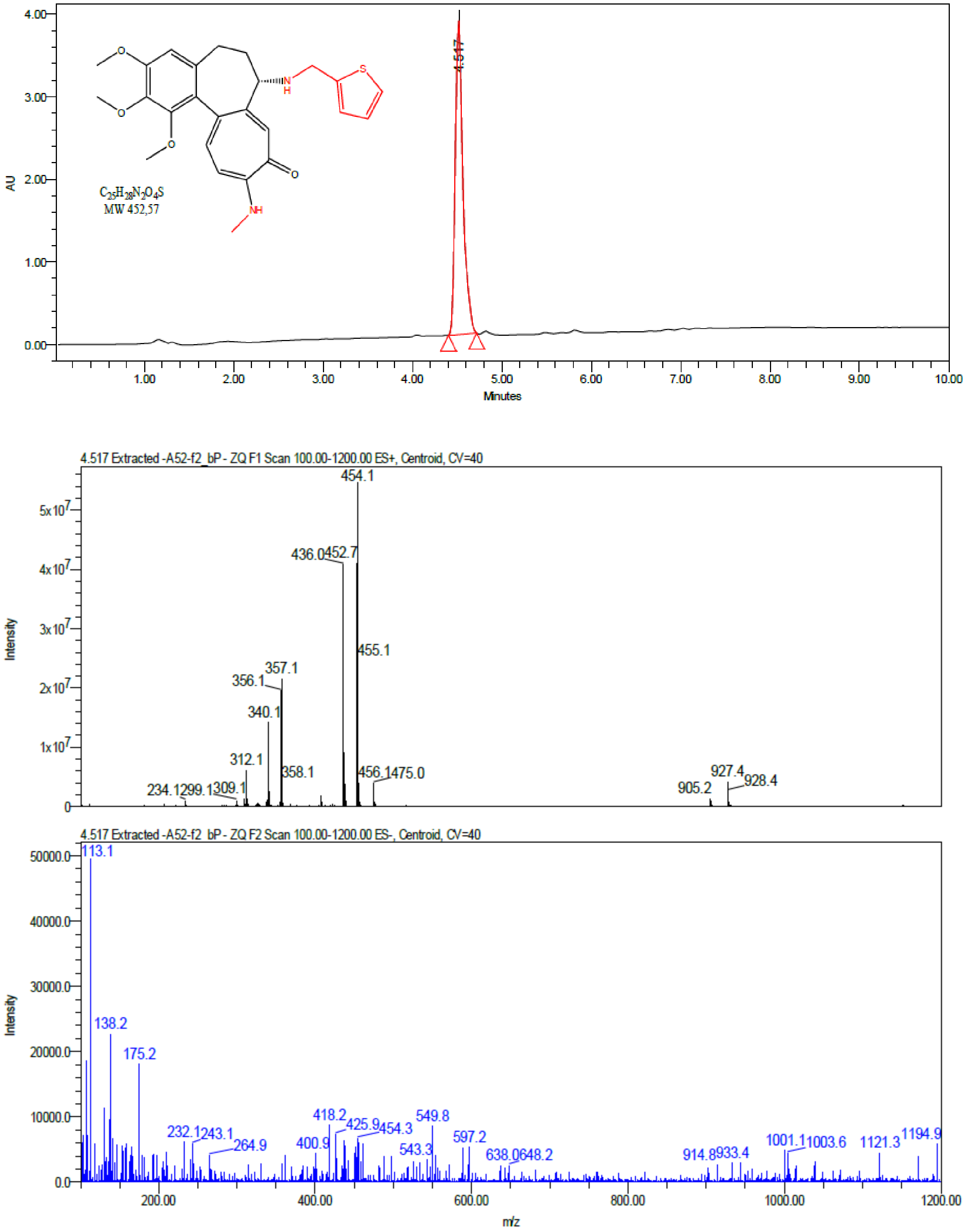

Figure S43. The LC-MS chromatogram and mass spectra of **17**.

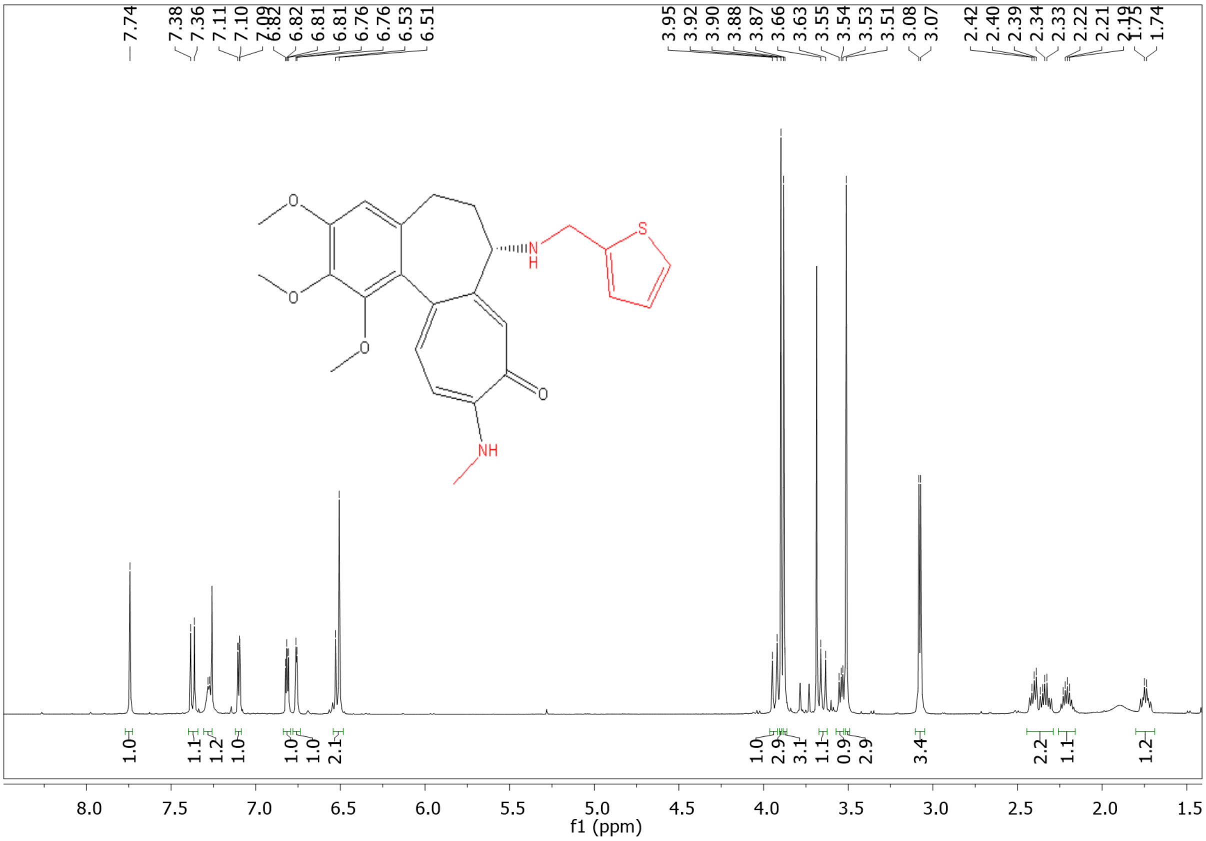

Figure S44. The ^{1}H NMR spectrum of **17** in $CDCl_3$.

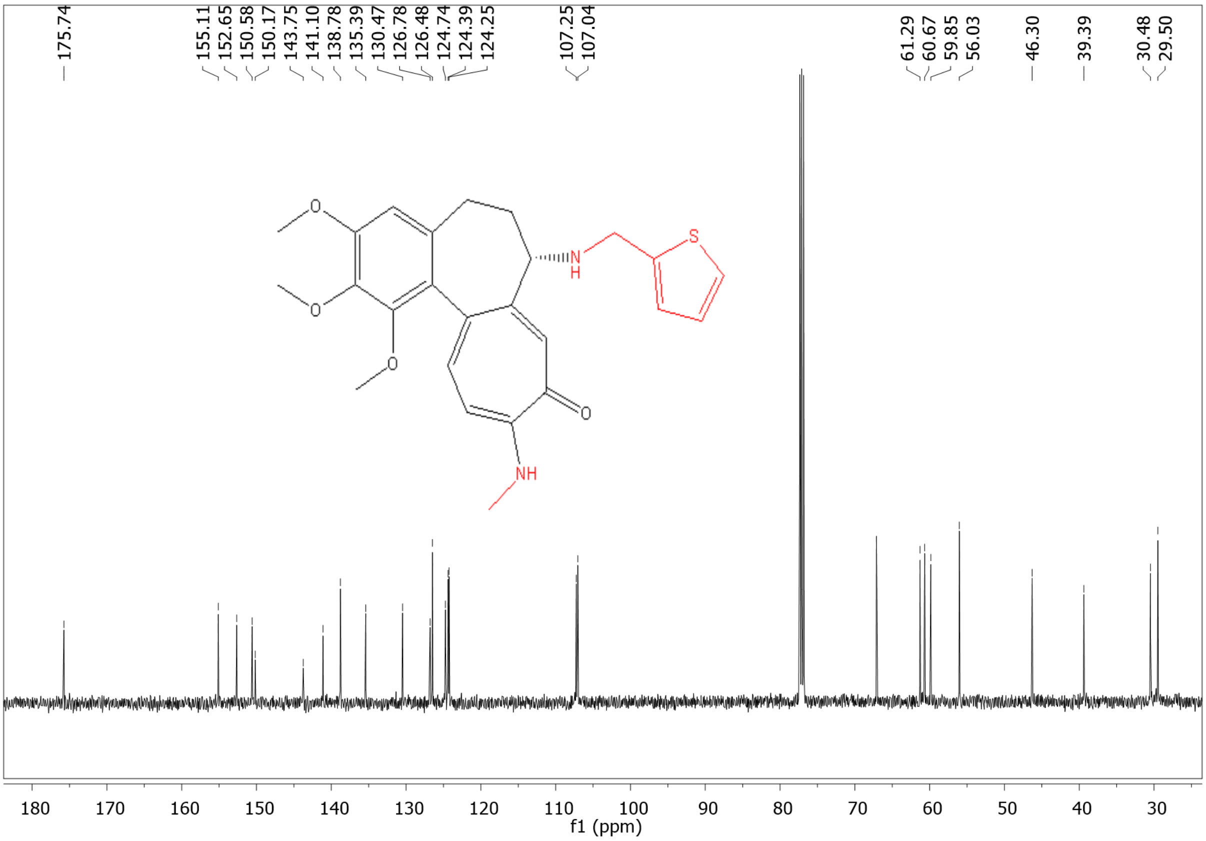

Figure S45. The ^{13}C NMR spectrum of **17** in $CDCl_3$.

1. Krzywik, J.; Mozga, W.; Aminpour, M.; Janczak, J.; Maj, E.; Wietrzyk, J.; Tuszyński, J.A.; Huczyński, A. Synthesis, antiproliferative activity and molecular docking studies of novel doubly modified colchicine amides and sulfonamides as anticancer agents. *Molecules* **2020**, *25*.

2. Skehan, P.; Storeng, R.; Scudiero, D.; Monks, A.; Mcmahon, J.; Vistica, D.; Warren, J.T.; Bokesch, H.; Kenney, S.; Boyd, M.R. New colorimetric cytotoxicity assay for anticancer-drug screening. *J. Natl. Cancer Inst.* **1990**, *82*, 1107–1112.

3. Nevozhay, D. Cheburator software for automatically calculating drug inhibitory concentrations from in vitroscreening assays. *PLoS One* **2014**, *9*, e106186.

4. Schrödinger Schrödinger Release 2019-4: LigPrep 2019.

5. Löwe, J.; Li, H.; Downing, K.H.; Nogales, E. Refined structure of αβ-tubulin at 3.5 Å resolution. *J. Mol. Biol.* **2001**, *313*, 1045–1057.

6. Molecular Operating Environment (MOE); Chemical Computing Group Inc.: Montreal, QC, Canada, 2012.

7. Case, DA. BR., Botello-Smith W., Cerutti DS., Cheatham TE. III, Darden TA., Duke RE., Giese TJ., Gohlke H., Goetz AW., Homeyer N., Izadi S., Janowski P., Kaus J., Kovalenko A., Lee TS., LeGrand S., Li P., Lin C., Luchko T., Luo R., Madej B., E. al. AMBER 2014 2014.

8. Roe, D.R.; Cheatham, T.E. PTRAJ and CPPTRAJ: Software for processing and analysis of molecular dynamics trajectory data. *J. Chem. Theory Comput.* **2013**, *9*, 3084–3095.

9. Trott, O.; Olson, A.J. Software news and update AutoDock Vina: Improving the speed and accuracy of docking with a new scoring function, efficient optimization, and multithreading. *J. Comput. Chem.* **2010**, *31*, 455–461.

10. Preto, J.; Gentile, F. Assessing and improving the performance of consensus docking strategies using the DockBox package. *J. Comput. Aided. Mol. Des.* **2019**, *33*, 817–829.

11. Hou, T.; Wang, J.; Li, Y.; Wang, W. Assessing the performance of the MM/PBSA and MM/GBSA methods. 1. The accuracy of binding free energy calculations based on molecular dynamics simulations. *J. Chem. Inf. Model.* **2011**, *51*, 69–82.

12. Sun, H.; Duan, L.; Chen, F.; Liu, H.; Wang, Z.; Pan, P.; Zhu, F.; Zhang, J.Z.H.; Hou, T. Assessing the performance of MM/PBSA and MM/GBSA methods. 7. Entropy effects on the performance of end-point binding free energy calculation approaches. *Phys. Chem. Chem. Phys.* **2018**, *20*, 14450–14460.
